# Diversity of Treponema denticola and Other Oral Treponeme Lineages in Subjects with Periodontitis and Gingivitis

**DOI:** 10.1128/Spectrum.00701-21

**Published:** 2021-09-29

**Authors:** Huihui Zeng, Yuki Chan, Wenling Gao, W. Keung Leung, Rory M. Watt

**Affiliations:** a Faculty of Dentistry, The University of Hong Kong, Hong Kong SAR, China; Shenzhen Bay Laboratory

**Keywords:** UMP kinase, etiology, bacterial infection, spirochetes, phylogeny, *Treponema denticola*, periodontitis, gingivitis, oral microbiome

## Abstract

More than 75 species/species-level phylotypes belonging to the genus Treponema inhabit the human oral cavity. Treponema denticola is commonly associated with periodontal disease, but the etiological roles and ecological distributions of other oral treponemes remain more obscure. Here, we compared the clinical distributions of phylogroup 1 and 2 oral treponemes in subgingival plaque sampled from Chinese subjects with periodontitis (*n* = 10) and gingivitis (*n* = 8) via sequence analysis of the highly conserved *pyrH* housekeeping gene. Two PCR primer sets that targeted oral phylogroup 1 and 2 treponeme *pyrH* genes were used to construct plasmid clone amplicon libraries for each subject, and the libraries were sequenced for bioinformatic analysis. A total of 1,204 quality-filtered, full-length *pyrH* gene sequences were obtained from the cohort (median number, 61.5 cloned *pyrH* sequences per subject; range, 59 to 83), which were assigned to 34 *pyrH* genotypes (designated pyrH001 to pyrH034; 97% sequence identity cutoff). Eighteen *pyrH* genotypes (536 *pyrH* sequences) corresponded to phylogroup 1 treponeme taxa (including Treponema vincentii and Treponema medium). Sixteen *pyrH* genotypes (668 *pyrH* sequences) corresponded to T. denticola and other phylogroup 2 treponemes. Samples from periodontitis subjects contained a greater diversity of phylogroup 2 *pyrH* genotypes than did samples from gingivitis subjects (Mann-Whitney U test). One T. denticola *pyrH* genotype (pyrH001) was highly prevalent, detected in 10/10 periodontitis and 6/8 gingivitis subjects. Several subjects harbored multiple T. denticola *pyrH* genotypes. Nonmetric multidimensional scaling and permutational multivariate analysis of variance (PERMANOVA) revealed no significant differences in overall *pyrH* genotype compositions between periodontitis and gingivitis subjects. Taken together, our results show that subjects with periodontitis and gingivitis commonly harbor highly taxonomically diverse communities of oral treponemes.

**IMPORTANCE** Periodontal diseases, such as periodontitis, are highly complex, multifactorial inflammatory infectious diseases affecting the gums and tooth-supporting structures. They are caused by chronic accumulations of dental plaque below the gum line that typically comprise hundreds of different bacterial species. Certain species of spiral-shaped bacteria known as treponemes, most notably Treponema denticola, are proposed to play key roles in the development and progression of periodontal disease. In our study, we characterized the genetic lineages of T. denticola, Treponema vincentii, Treponema medium, and related species of treponeme bacteria that were present in dental plaque samples from Chinese subjects with periodontal disease. Our results revealed that individual subjects commonly harbored multiple genetic lineages (strains) of T. denticola and other species of treponeme bacteria. Taken together, our results indicate that highly diverse and complex populations of oral treponemes may be present in dental plaque, which may potentially play important roles affecting periodontal health status.

## INTRODUCTION

Bacterial taxa belonging to the genus Treponema (commonly referred to as treponemes) are the only spirochete taxa known to commonly colonize the human oral cavity. They typically inhabit oxygen-depleted niches, especially dental plaque biofilms found within the gingival sulcus, the shallow crevice of gum tissue that surrounds the base of the tooth (1, 2). Treponemes constitute a relatively small (e.g., <2%) but significant and taxonomically diverse proportion of the healthy human oral microbiome (3, 4). However, it has been known for decades that their proportions are commonly highly elevated within chronically infected subgingival sites found in individuals with periodontal diseases, such as gingivitis and periodontitis (see below) (5–10).

There are an estimated 75 to 100 species-level phylotypes of human oral treponeme bacteria, the majority of which have been identified solely by molecular methods (4, 11, 12). Oral treponemes have been phylogenetically classified into 10 oral phylogroups (numbered 1 to 10) (11, 13) and *ca*. 50 human microbiome taxon (HMT) groups (4, 14) based on levels of shared 16S rRNA gene sequence similarity. There are 9 formally classified Treponema species of human oral origin (oral treponeme phylogroups in parentheses): Treponema medium (1), Treponema denticola (2), Treponema putidum (2), Treponema lecithinolyticum (4), Treponema maltophilum (4), Treponema amylovorum (5), Treponema socranskii subsp. *socranskii*, *paredis*, *buccale*, and 04 (6), Treponema parvum (7), and Treponema pectinovorum (8). Treponema vincentii (phylogroup 1) is commonly reported in the scientific literature but remains to be formally reported (15, 16). Three other species-level phylotypes of T. medium-like or T. vincentii-like phylogroup 1 treponemes have been identified (named Treponema species IA, IB, and IC) based on results from a multilocus sequence analysis (MLSA) of four highly conserved genes: 16S rRNA (rRNA), *recA* (recombinase A), *pyrH* (uridylate kinase), and *flaA* (flagellar sheath protein) (17).

The term “periodontal disease” encompasses a spectrum of related inflammatory infectious diseases affecting the tissues that surround and support the teeth (18). Periodontal diseases range from milder forms, i.e., gingivitis, in which the inflammation is limited to the soft tissue components of the periodontium, to more serious forms, i.e., periodontitis, in which the underlying bone tissues are also affected (19). They are highly prevalent in global populations, and severe periodontitis is the major cause of tooth loss in adults (20). The clinical classification of periodontal diseases has recently been revised and updated. Periodontitis is now characterized based on a multidimensional staging system (stage I, initial periodontitis; stage II, moderate periodontitis; stage III, severe periodontitis; and stage IV, advanced periodontitis), as well as a grading system (grade A, low risk; grade B, moderate risk; and grade C, high risk) (21, 22). Gingivitis is clinically assessed using percentage of full-mouth bleeding on probing (%BOP) scores according to a dichotomized scale, i.e., at ≥10% BOP without attachment loss or radiographic bone loss or having a reduced periodontium without a history of periodontitis or with prior successful treatment (23). The reduced-periodontium category may experience attachment loss and, hence, radiographic bone loss.

Periodontal disease has a complex polymicrobial etiology that is typified by elevated populations of proteolytic and anaerobic bacterial species in subgingival plaque biofilm communities. There is a large body of evidence linking Treponema denticola with severe forms of periodontal disease (5, 24–30). Molecular studies performed over the past 10 to 20 years have associated several other oral treponeme species with periodontal disease. Several studies have implicated species or phylotypes (HMTs) corresponding to T. medium, T. vincentii, or closely related oral phylogroup 1 treponeme taxa with the etiology of periodontitis (10, 29, 31–35). However, uncertainties in taxonomy within this cluster of species/phylotypes hinder accurate etiological associations (11, 12, 15).

Here, we analyzed *pyrH* gene sequences carried by oral phylogroup 1 and 2 treponeme bacteria present in human subgingival plaque samples collected from subjects with gingivitis (*n *= 8) versus subjects with severe forms of periodontitis (*n *= 10). Our results showed that both gingivitis and periodontitis subjects harbored diverse communities of phylogroup 1 and 2 treponemes, as indicated by the respective sets of *pyrH* gene sequences identified. Certain phylogenetic lineages of *pyrH* sequences, including one closely related to T. denticola ATCC 35405^T^, were highly prevalent within both gingivitis and periodontitis subject groups. Other treponeme *pyrH* gene lineages showed disease-selective distributions. In summary, our results demonstrate that individuals with both milder and more severe forms of periodontal disease may harbor multiple genetic lineages corresponding to the same species or species-level phylotype of oral treponeme.

## RESULTS

### Clinical evaluation of periodontal status in the two subject groups.

Eighteen adult subjects with periodontitis (P; *n *= 10) or gingivitis (G; *n *= 8) were recruited with informed consent. Their respective demographic profiles and full-mouth clinical parameters are summarized in Tables 1 and 2. Three of the P subjects were classified as having stage IV grade C periodontitis, six P subjects as having stage III grade C periodontitis, and one P subject as having stage III grade B periodontitis according to the recently revised classification system (Table 2) (20, 21). The mean age of P group subjects (35.8 ± 9.6 years [mean ± standard deviation]) was significantly younger than that of the G group (49.5 ± 5.3 years) (*P = *0.002 by independent-samples *t* test). Consistent with their disease status, the P group subjects (76.4% ± 16.9%) had significantly higher numbers of sites that bled on gentle probing (reported as full-mouth bleeding on probing [BOP] scores) compared to the G group (30.4% ± 18.4%) (*P < *0.0001 by independent-samples *t* test).

**TABLE 1:** Summary of demography and clinical parameters within periodontitis and gingivitis subject groups

Parameter^*a*^	Type of value	Periodontal condition		Statistical test	*P* value^*c*^
		Periodontitis (*n *= 10)	Gingivitis (*n *= 8)^*b*^		
Profile of subjects					
Age (yr)	Mean ± SD	35.8 ± 9.6	49.5 ± 5.3	*t* test	**0.002**
Gender	No. of males	7	3	Fisher’s exact *t* test	0.45
	No. of females	3	5		
Standing teeth	Median no.	27	26	Mann-Whitney U test	0.633
	Interquartile range	3	2		
Full-mouth BOP score (%)	Mean ± SD	76.4 ± 16.9	30.4 ± 18.4	*t* test	**<0.0001**
	Range	63.6–100	12.4–60.7		
Full-mouth PPD ≥5 mm (%)	Mean ± SD	20.8 ± 17.10	0	*t* test	**0.004**
	Range	2.7–52.3	0		

Profile of sampled sites					
No. sampled	Mean ± SD	32.0 ± 24.6	157.8 ± 7.5	*t* test	**<0.0001**
	Range	4–69	144–168		
BOP (%)	Median	99.3	21.5	Mann-Whitney U test	**<0.0001**
	Interquartile range	6.0	33.0		
Mean PPD (mm)	Mean ± SD	5.9 ± 0.4	ND		
	Range	5.3–6.5	ND		

a PPD, pocket-probing depth; BOP, bleeding on probing.

b The PPD values for all gingivitis group subjects were ≤3 mm according to the criteria for the group and were not recorded. ND, not determined.

c *P* values of <0.05 are indicated with boldface.

**TABLE 2:** Summary of demography, clinical parameters, and periodontal status for each subject

Subject	Gender^*a*^	Age (yr)	Full-mouth periodontal profile			Profile of sampled sites			Periodontal status^*b*^	
			No. of standing teeth	%BOP	%PPD ≥5 mm	No. of sites	%BOP	Mean PPD (mm)	Stage	Grade or clinical assessment
P1	M	28	28	79.8	38.7	65	100.0	6.3	IV	C**
P2	F	44	25	91.3	37.3	56	100.0	6.2	IV	C**
P3^*c*^	M	54	22	99.2	52.3	69	98.6	6.4	IV	C*
P4	M	31	27	91.4	28.4	46	93.5	5.5	III	C*
P5	M	26	24	56.3	5.6	8	100.0	5.9	III	C*
P6	M	23	28	72.6	12.5	21	95.2	5.4	III	C*
P7	M	37	27	92.0	14.2	23	100.0	6.0	III	C*
P8	F	39	28	62.5	10.1	17	94.1	6.0	III	C*
P9	F	44	25	68.0	2.7	4	100.0	5.3	III	C*
P10	M	32	28	50.6	6.5	11	63.6	5.6	III	B*
G1	F	47	27	50.0	0.0	162	50.0	ND^*d*^	—	##
G2	F	55	26	44.2	0.0	156	44.2	ND	—	##
G3	M	51	25	60.7	0.0	150	60.7	ND	—	##
G4	M	50	27	12.3	0.0	162	12.3	ND	—	#
G5	F	50	24	14.6	0.0	144	14.6	ND	—	#
G6	F	54	26	19.9	0.0	156	19.9	ND	—	#
G7	M	51	28	18.5	0.0	168	18.5	ND	—	#
G8^*c*^	F	38	26	23.1	0.0	156	23.1	ND	—	#

a M, male; F, female.

b Stage and grade apply to periodontitis and are further described in the text. **, generalized periodontitis (≥30% PPD ≥5 mm); *, localized periodontitis (<30% PPD ≥5 mm); —, not applicable; ##, generalized gingivitis (≥30% BOP); #, localized gingivitis (<30% BOP).

c Current smoker.

d ND, not determined due to clinical classification of gingivitis (see Table 1).

After clinical examination, a pooled, multisite sample of subgingival plaque was carefully collected from each subject for nucleic acid purification and molecular analysis. The respective numbers of sites sampled for each subject, as well as the corresponding BOP scores (%) and probing pocket depths (PPD, in mm) for the sampled sites, are summarized in Tables 1 and 2. In the P subjects, the median BOP for sampled sites was 99.3%, and the mean PPD and standard deviation for the sampled sites was 5.9 ± 0.4 mm. This indicated that the vast majority of the sampled sites corresponded to clinically diseased sites. In the G subjects, *ca*. one-fifth of the sampled sites tested positive for BOP, indicating that subgingival plaque was collected from a mixture of clinically diseased and nondiseased (clinically asymptomatic) sites.

### Identification of clinical *pyrH* genotypes present within subgingival niches in the two groups.

Two sets of PCR primers were used to amplify full-length *pyrH* gene sequences from oral phylogroup 1 and 2 treponemes present in the subgingival plaque samples collected from each subject. These two PCR primer sets have previously been shown to specifically amplify *pyrH* gene sequences from a diverse set of treponeme isolates belonging to oral phylogroups 1 and 2 (17). PCR amplicons of *ca*. 700 bp were successfully obtained using both primer sets for each subject (Table S1). The 36 PCR amplicons were cloned into TOPO (pCR2.1) plasmids to create TOPO plasmid clone libraries of phylogroup 1 and 2 *pyrH* genes for each subject. A subset of the resultant plasmid clones obtained from each of the 36 libraries (range, 26 to 53) were sequenced bidirectionally. A total of 1,204 quality-filtered, full-length *pyrH* gene sequence reads (herein described as “cloned *pyrH* sequences”) were recovered from the clinical cohort (Table S1). A total of 536 cloned *pyrH* sequences were obtained using the oral treponeme phylogroup 1 primer set, and 668 cloned *pyrH* sequences were obtained using the oral treponeme phylogroup 2 primers. The specificity was 100%, with no off-target sequences detected. All of the phylogroup 1 *pyrH* gene amplicons were 687 bp in length (average G+C content, 46.0% ± 1.2%), encoding (putative) PyrH proteins of 228 amino acids (aa). All phylogroup 2 *pyrH* gene amplicons were 696 bp in length (average G+C content, 40.8% ± 0.7%), encoding PyrH proteins 231 aa in length.

The *pyrH* gene sequences (*n *= 1,204) were clustered into 34 *pyrH* genotypes, based on a 97% (average neighbor) sequence identity cutoff (Fig. S1 and Table S2). This cutoff value was used based on results from a previous study of *pyrH* gene sequence diversity in a collection of clinical isolates (and reference strains) that encompassed seven different oral treponeme species or species-level phylotypes that belonged to oral treponeme phylogroups 1 and 2 (see Discussion) (17). These 34 *pyrH* genotypes were named pyrH001 to pyrH034 according to the total number of cloned *pyrH* gene sequences that corresponded to each genotype.

The numbers of *pyrH* genotypes identified within the P and G subject groups are summarized in Table 3. There was no significant difference in the median numbers of cloned *pyrH* gene sequences obtained from the P and G groups (median = 61.5 for both groups), nor for the number of cloned *pyrH* sequences obtained using the phylogroup 1 or 2 PCR primer sets (*n *= 30 or 31.5 for the two groups). There was no significant difference in the median numbers of phylogroup 1 *pyrH* genotypes identified in the P and G groups (*n *= 3.5 and *n *= 3, respectively). However, the median number of phylogroup 2 *pyrH* genotypes identified in the P group (*n *= 3) was higher than the median number identified within the G group (*n* = 1.5) (*P = *0.009, Mann-Whitney U test) (Table 3).

**TABLE 3:** Summary of *pyrH* genotypes and numbers of cloned *pyrH* gene sequences recovered from the periodontitis and gingivitis subject groups

Parameter	Median no. (range) in group with:		*P* value^*a*^
	Periodontitis	Gingivitis	
*pyrH* genotypes identified	7 (4–12)	5 (4–7)	**0.034**
Cloned *pyrH* gene sequences obtained	61.5 (59–81)	61.5 (55–83)	1.000
Phylogroup 1 *pyrH* genotypes identified	3.5 (2–6)	3 (3–4)	0.696
Cloned *pyrH* gene sequences corresponding to phylogroup 1 treponeme species/phylotypes	30 (29–30)	30 (29–30)	0.573
Phylogroup 2 *pyrH* genotypes identified	3 (2–6)	1.5 (1–3)	**0.009**
Cloned *pyrH* gene sequences corresponding to phylogroup 2 treponeme species/phylotypes	31.5 (30–52)	31.5 (26–53)	0.829

a Mann-Whitney U test. *P* values of <0.05 are indicated with boldface.

**FIG 1:**
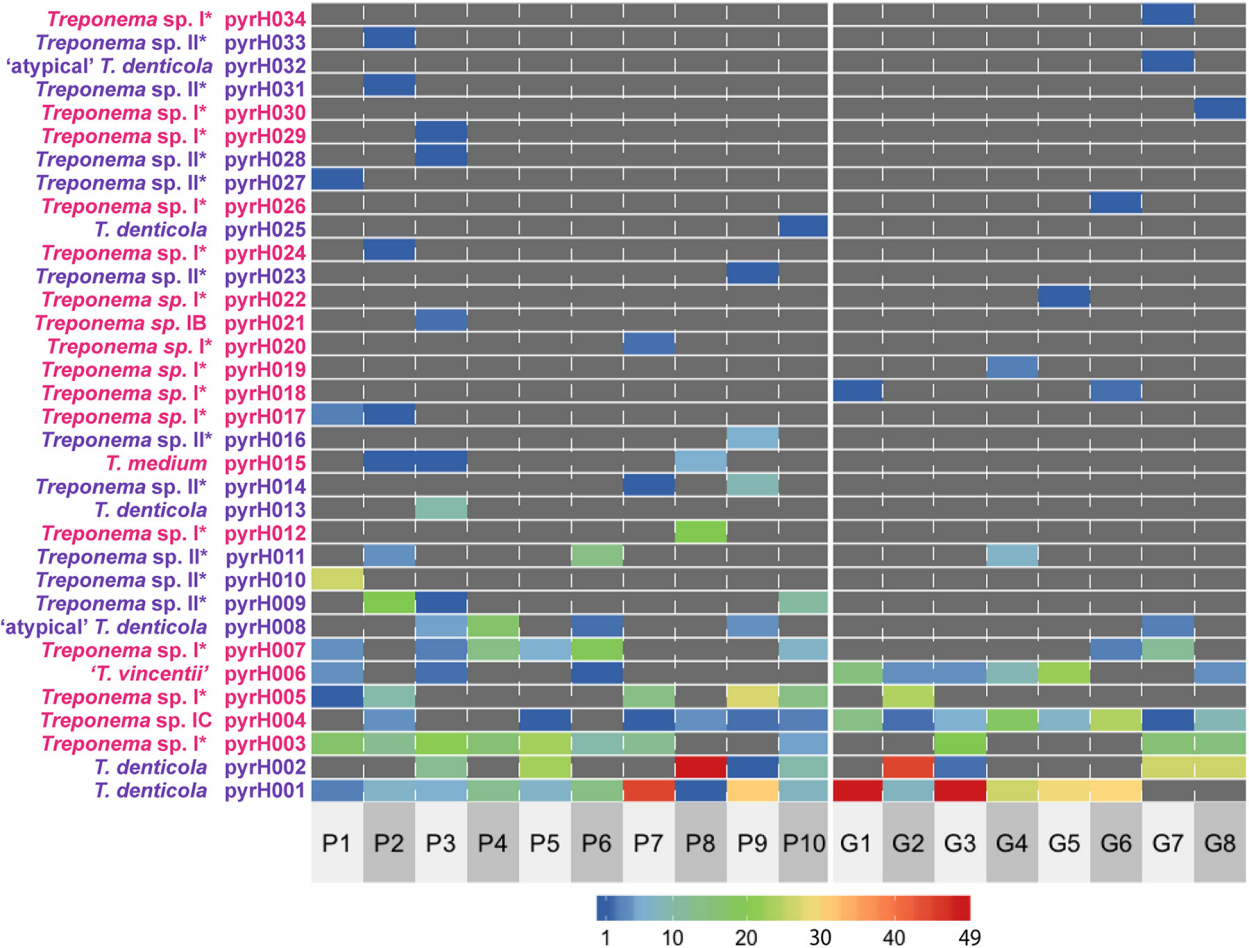
Heat map showing the clonal abundances of the 34 oral treponeme *pyrH* genotypes detected within the oral cavity of each subject. The designations of the 34 treponeme *pyrH* genotypes (pyrH001 to pyrH034) are shown on the *y* axis. The *pyrH* genotypes that correspond to oral phylogroup 2 taxa are shown in purple, and those that correspond to phylogroup 1 are shown in pink. The identifiers of the 18 subjects are indicated below the *x* axis (P1 to P10, periodontitis group; G1 to G8, gingivitis group). The clonal abundance of each treponeme *pyrH* genotype (i.e., the number of cloned *pyrH* gene sequences assigned to each *pyrH* genotype) in each subject is represented by color according to the scale bar shown below the heat map, in values ranging from 1 (dark blue) to 49 (red). Zero values are colored dark gray.

**FIG 2:**
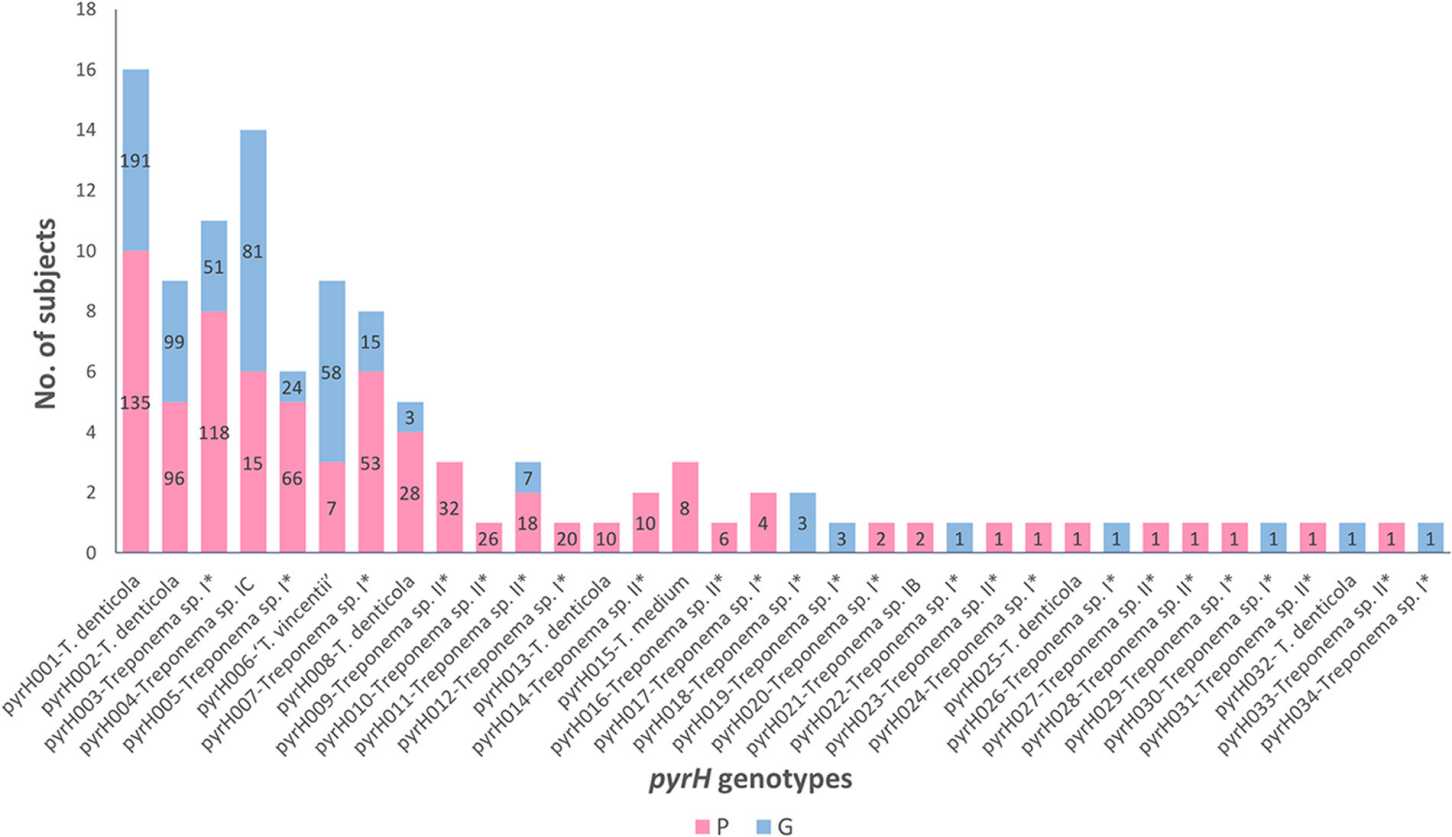
Prevalences of the oral treponeme *pyrH* genotypes detected in the periodontitis (P) and gingivitis (G) subject groups. The heights of the bars indicate the detection frequencies of the *pyrH* genotypes (pyrH001 to pyrH034) in the P and G subject groups (i.e., indicate the total number of subjects that tested positive for each *pyrH* genotype). The colors represent the prevalences within the subject groups as shown below the graph. The numbers in the bars indicate the clonal abundances of the 34 *pyrH* genotypes (i.e., the corresponding number of cloned *pyrH* gene sequences) in the P and G subject groups (out of a total of 1,224 cloned *pyrH* gene sequences). Thirteen *pyrH* genotypes (pyrH022 to pyrH034) were identified by a single cloned *pyrH* gene sequence.

A total of 326 cloned *pyrH* gene sequences corresponded to the pyrH001 genotype, making it the most frequently detected genotype within the cohort (Fig. 1 and Table S2). PyrH001 was the most frequently detected genotype in both the P subjects (*n *= 135) and the G subjects (*n *= 191; discussed further below). Thirteen *pyrH* genotypes (pyrH022 to pyrH034) were each represented by a single cloned *pyrH* sequence (Fig. 2).

The alpha diversity and richness of the respective *pyrH* genotypes identified within the two clinical groups were determined using the Chao1, ACE (abundance-based coverage estimator), Shannon, Simpson’s, and Good’s coverage estimators (summarized in Tables S3 and S4). All individuals were sampled adequately as indicated by Good’s coverage values, which ranged from 0.9 to 1.0 for both the phylogroup 1 and 2 *pyrH* genotypes.

### Taxonomic classification and phylogenetic relationships between *pyrH* genotypes.

**FIG 3:**
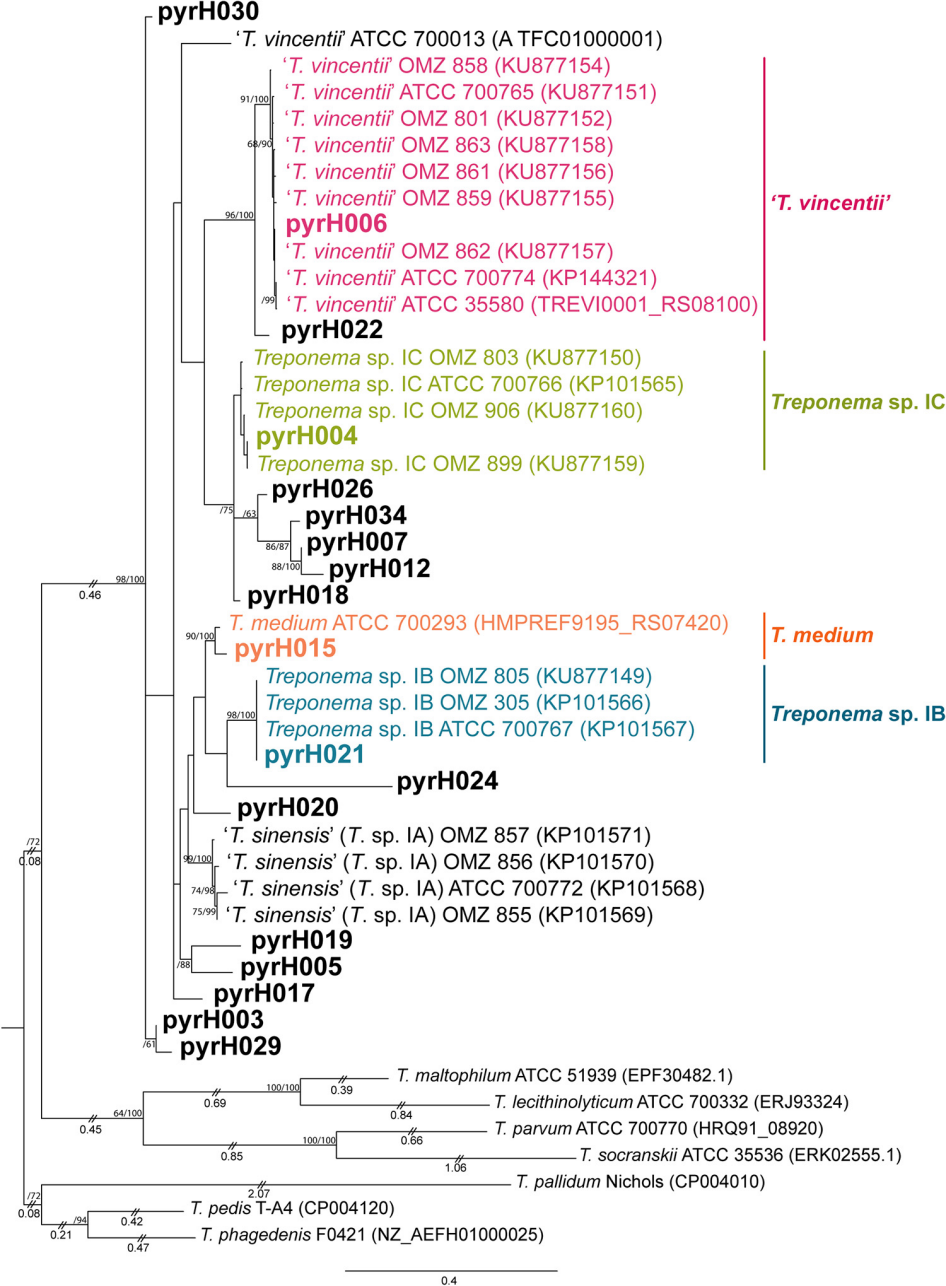
Maximum-likelihood (ML) phylogenetic tree of *pyrH* genes from oral treponeme phylogroup 1 taxa. The maximum clade credibility tree topology is supported by bootstrapping for 1,000 replicates (first number) and Bayesian posterior probabilities (second number) reported as percentage values separated with a forward slash (“/”) beside the branch nodes. Only values over 60% are shown. Treponeme genotypes identified in this study are indicated in boldface. Excessively long branches have been trimmed in proportion to the scale bar (indicated with double slashes [“//”] on the respective branches). The scale bar indicates 0.4 nucleotide changes per position. The oral treponeme species (phylogroups) are indicated with different colors as follows: *T. vincentii*, pink; *T. medium*, orange; Treponema sp. IC, yellow-green, and Treponema sp. IB, cyan. Treponema pedis T-A4, Treponema phagedenis F0421, Treponema pallidum Nichols, Treponema socranskii subsp. *socranskii* ATCC 35536, Treponema parvum ATCC 700770, Treponema maltophilum ATCC 51939, and Treponema lecithinolyticum ATCC 700332 were included as outgroup species.

**FIG 4:**
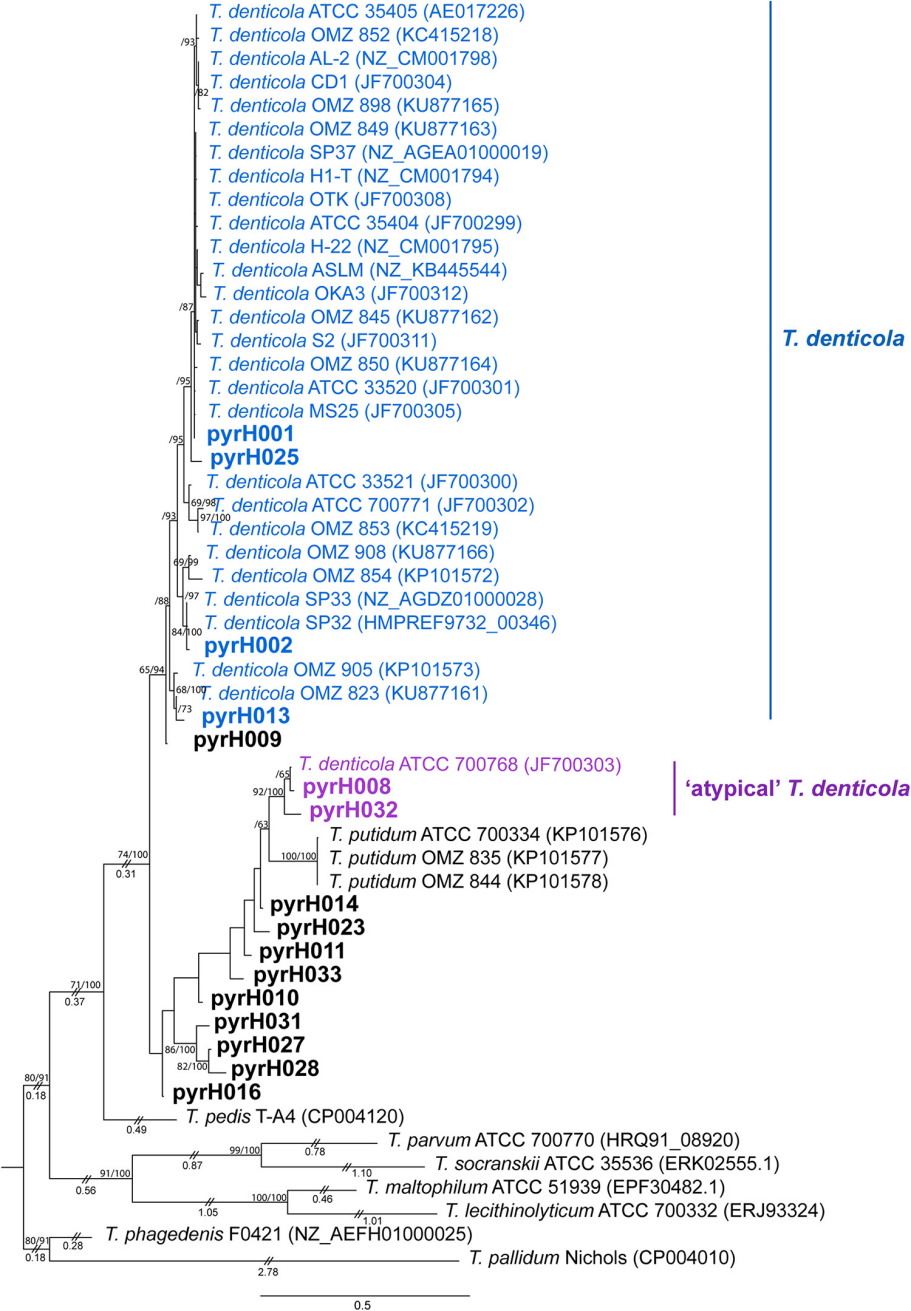
Maximum-likelihood phylogenetic tree of *pyrH* genes from oral treponeme phylogroup 2 taxa. The respective oral treponeme species (phylogroups) are indicated with different colors as follows: T. denticola, blue, and atypical T. denticola strains, purple. The scale bar indicates 0.5 nucleotide changes per position. For other explanatory details, refer to the legend to Fig. 3.

Phylogenetic relationships between the 34 *pyrH* genotypes were inferred by maximum-likelihood (ML) estimation (Fig. 3 and 4) with reference to results from a previous study (17). In our analysis, we included *pyrH* gene sequence data from several oral treponeme strains whose genomes have been deposited in the eHOMD database (14). The ML analyses included 22 oral phylogroup 1 and 31 oral phylogroup 2 treponeme strains. Full-length *pyrH* sequences from Treponema lecithinolyticum ATCC 700332^T^ (phylogroup 4), Treponema maltophilum ATCC 51939^T^ (phylogroup 4), Treponema parvum ATCC 700770^T^ (phylogroup 7), and Treponema socranskii subsp. *socranskii* ATCC 35536 (phylogroup 6) were included as outgroups. The best substitution model used for the ML trees was GTR+G+I, as determined by jModeltest2 (36).

Eighteen of the 34 clinical *pyrH* genotypes were classified as phylogroup 1 treponeme taxa (Fig. 3), with 16 corresponding to phylogroup 2 treponemes (Fig. 4). Twelve of the 34 *pyrH* genotypes could be taxonomically assigned to a previously identified oral treponeme species or phylotype (represented by different colors in Fig. 3 and 4; Table S2) (17). The other 22 *pyrH* genotypes were classified as corresponding to oral phylogroup 1 treponemes of uncertain taxonomic standing (Treponema sp. I*) or to oral phylogroup 2 treponemes of uncertain taxonomic standing (Treponema sp. II*) (shown in black in Fig. 3 and 4).

### Phylogroup 2 oral treponemes, including T. denticola and *T. putidum*.

Four *pyrH* genotypes (pyrH001, pyrH002, pyrH013, and pyrH025) corresponded to T. denticola (colored blue in Fig. 4). Genotype pyrH001 was located in a large clade containing the type strain ATCC 35405 (Fig. 4). Genotype pyrH002 was most closely related to the SP33 and SP32 strains (mean identity, 99.3%), while pyrH013 formed a distinct clade with the OMZ 823 and OMZ 905 strains (mean identity, 97.8%).

Genotypes pyrH008 and pyrH032 formed a distinct clade with the ATCC 700768 (OMZ 830) strain, which was originally classified (and deposited) as an atypical strain of T. denticola (mean identity, 97.6%) (shown in purple in Fig. 4 and discussed further below) (37). The *pyrH* gene sequence of this atypical strain is more closely related to those from isolates of T. putidum than from the other T. denticola strains sequenced to date. Genotypes pyrH027, pyrH028, and pyrH031 formed a distinct clade (within-clade mean identity, 95%) that was well separated from the other phylogroup 2 taxa. None of the clinical *pyrH* genotypes identified within the cohort corresponded to T. putidum, which is the only other formally characterized species in phylogroup 2. Furthermore, we did not detect any *pyrH* gene sequences corresponding to closely related (pathogenic, mammalian host-associated) treponeme species, such as Treponema phagedenis, Treponema pallidum, or Treponema pedis (Fig. 4 and Fig. S2).

### Phylogroup 1 oral treponemes, including *T. medium* and *T. vincentii*.

We identified *pyrH* genotypes corresponding to 4 of the 5 previously classified species/species-level phylotypes of oral phylogroup 1 treponemes (17, 38), including T. vincentii (pyrH006), T. medium (pyrH015), Treponema sp. IB (pyrH021), and Treponema sp. IC (pyrH004). We did not detect Treponema sp. IA (Treponema sinensis) (17) in this subject group. It may be noted that the sequence of the *pyrH* gene from the ATCC 700013 (OMZ 779) strain of T. vincentii is highly anomalous compared to the sequences of the *pyrH* genes carried by all other known strains in this species (17). We identified seven *pyrH* genotypes that were divergent from those of the previously characterized oral phylogroup 1 taxa, including two that were prevalent in the cohort and were detected with high frequency (pyrH003 and pyrH007; see Discussion) (Fig. 1). The sequence of the pyrH021 genotype was identical to that of the *pyrH* gene present in all 3 known strains of Treponema sp. IB, strains OMZ 305 (which was formerly classified as T. vincentii Ritz A), OMZ 805, and ATCC 700767 (OMZ 806) (17). The pyrH017, pyrH020, pyrH022, and pyrH024 genotypes represented distinct single-taxon lineages. The pyrH005 and pyrH019 genotypes formed a distinct, well-separated clade. The pyrH030 genotype was a notable outlier and was phylogenetically divergent from all other known *pyrH* gene sequences.

### Distributions of *pyrH* genotypes in the two clinical groups.

The absolute counts of cloned *pyrH* gene sequences within each of the respective subjects were visualized in a heat map (Fig. 1). The prevalence of each *pyrH* genotype within the P and G clinical groups (i.e., detection/nondetection in each subject) is shown in a bar chart in Fig. 2. The T. denticola pyrH001 genotype was the most prevalent *pyrH* genotype in the cohort, found in 10/10 of periodontitis subjects and 6/8 gingivitis subjects. Although its prevalence was slightly higher in the P group, a higher number of cloned *pyrH* gene sequences was obtained from the G group subjects (*n *= 191 versus *n *= 135) (Fig. 2). The T. denticola pyrH002 genotype was also prevalent in the cohort, being detected in 5/10 P subjects and 4/8 G subjects, with approximately equal numbers of cloned *pyrH* sequences obtained from P versus G subjects. Genotype pyrH003 (Treponema phylogroup I*) was highly prevalent in the P group subjects (8/10) and less prevalent in the G subjects (3/8). Conversely, the Treponema sp. IC pyrH004 and T. vincentii pyrH006 genotypes were highly prevalent in the G subjects (8/8 and 6/8, respectively) but less prevalent in the P subjects (6/10 and 3/10, respectively).

**FIG 5:**
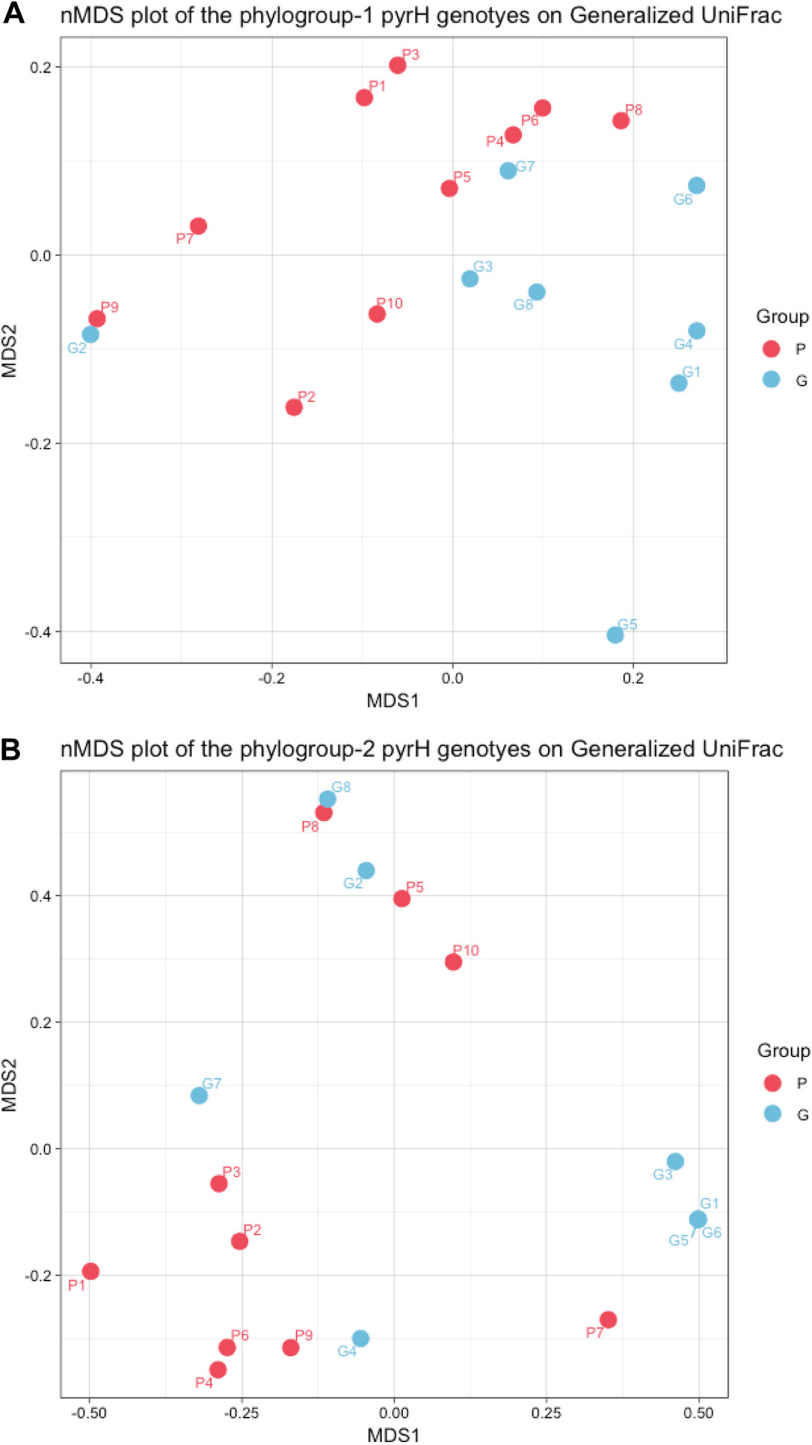
Nonmetric multidimensional scaling (nMDS) analysis of oral treponeme *pyrH* genotypes present within each subject. The nMDS analysis was performed using a distance matrix calculated using a generalized UniFrac algorithm based on a single maximum-likelihood (ML) tree containing all treponeme *pyrH* genotype sequences detected (*n *= 34). The dots represent the 10 periodontitis subjects (P1 to P10) and the 8 gingivitis subjects (G1 to G8) by color as shown to the right of the plots. (A) Plot showing the nMDS of treponeme phylogroup 1 genotypes (*R*^2^ = 0.157, *P = *0.018). (B) Plot showing the nMDS of treponeme phylogroup 2 genotypes (*R*^2^ = 0.114, *P = *0.097).

Nonmetric multidimensional scaling (nMDS) ordination analyses of the compositions of clinical *pyrH* phylogroup 1 and 2 genotypes based on the generalized UniFrac distance are shown in in Fig. 5A and B. The segregation between the *pyrH* genotypes from the P and G groups is more apparent in the phylogroup 1 data. However, permutational multivariate analysis of variance (PERMANOVA) tests indicated that the differences between the two clinical groups were not significant (PERMANOVA overall *R*^2^ = 0.099, *P = *0.121; phylogroup 1 *R*^2^ = 0.157, *P = *0.018; phylogroup 2 *R*^2^ = 0.114, *P = *0.097).

## DISCUSSION

Here, we analyzed differences in treponeme populations within subgingival plaque biofilms sampled from human subjects with gingivitis and periodontitis, using the *pyrH* gene as a diagnostic genetic biomarker. We had two major aims. The first aim was to determine if there were notable differences in the distributions of T. denticola, T. vincentii, T. medium, and closely related oral treponeme phylotypes within patients with periodontitis versus those suffering from gingivitis. The second aim was to establish whether or not subjects with periodontitis or gingivitis harbored multiple genetic lineages (i.e., different clinical strains) of the same treponeme species/phylotype within their respective oral cavities.

We focused on phylogroup 1 and 2 oral treponemes for the following reasons. Numerous studies have associated T. denticola with periodontal disease, and an assortment of virulence factors have been identified for this species (28, 29, 39–41). However, T. putidum is phenotypically and phylogenetically very similar to T. denticola, and yet, it appears to be relatively rarely detected in clinical cohorts (10–12, 37, 42, 43). In addition, the etiological roles of commonly detected phylogroup 1 oral treponeme species (phylotypes) like T. vincentii and T. medium have yet to be firmly established (5, 12, 15, 41, 44).

The *pyrH* gene was selected as it has previously been shown to have good strain-resolving abilities, as well as a robust correlation with the phylogeny of the 16S rRNA gene within clinical isolates of T. denticola, T. putidum, T. vincentii, T. medium, and other species-level phylotypes within oral treponeme phylogroups 1 and 2 (17, 45). This highly conserved, single-copy housekeeping gene has previously been used to assign taxonomy and to infer phylogenetic relationships in MLSA studies conducted within other bacterial species, including *Vibrio* spp. (46) and *Photobacterium* spp. (47). Our sequencing data show that the two *pyrH* PCR primer sets used each had excellent specificity, with no off-target hits detected. Due to the limited number of oral treponeme genomes published to date, it is difficult to accurately gauge their clinical detection range. However, they were previously shown to successfully amplify *pyrH* gene amplicons from all clinical strains tested (17, 45), which validates their effectiveness. Our use of a focused TOPO clone library sequencing approach offers the advantage of generating high-quality, full-length *pyrH* gene sequences for detailed phylogenetic and taxonomic evaluation. However, this comes at the expense of excluding the majority of Treponema taxa and losing quantitative information that may be retained using alternative sequencing approaches (see below).

Our results clearly demonstrate that individuals with periodontitis or gingivitis commonly harbor multiple lineages of the same Treponema species/phylotype within their respective oral cavities. For example, the oral cavities of subjects P3, P9, and P10 each contained at least 3 distinct *pyrH* genotypes that corresponded to T. denticola (Fig. 1).

The most prevalent and frequently detected *pyrH* genotype (pyrH001) corresponded to a large cluster of previously identified T. denticola isolates that includes the ATCC 33520 and ATCC 35405^T^ strains (Fig. 4). Strains within this cluster were originally isolated from subjects who resided in various countries across Asia, Europe, and North America (17, 45). The second most prevalent *pyrH* genotype (pyrH002) was phylogenetically most closely related to two North American T. denticola strains (SP32 and SP33) whose genomes were sequenced at the Broad Institute and subsequently deposited in the NCBI GenBank (SP32, GCA_000413095.1, and SP33, GCA_000338475.1; unpublished). Taken together, this suggests that there are several T. denticola lineages (clinical strains) that are highly prevalent within global populations.

Our results showed that subgingival plaque samples collected from periodontitis subjects contained a greater detectable diversity of phylogroup 1 and 2 oral treponeme *pyrH* genotypes than corresponding samples from gingivitis subjects (i.e., a higher median number of *pyrH* genotypes were detected in P subjects) (Table 3). In particular, the diversity of phylogroup 2 oral treponemes was significantly higher in periodontitis subjects than in gingivitis subjects. This is consistent with results from a previous 16S rRNA gene amplicon-based analysis of oral treponemes in subjects with or without periodontitis (12). In this previous study, periodontitis subjects had significantly higher levels of phylogroup 2 treponeme operational taxonomic unit (OTU) richness and clonal abundance, with one T. denticola lineage (OTU 8P47) having a statistically significant association with periodontitis. Many of the phylogroup 2 *pyrH* genotypes we detected here correspond to as-yet-uncharacterized genetic lineages of uncertain taxonomic standing (Fig. 4). Several of these almost certainly correspond to as-yet-uncharacterized lineages of T. denticola; however, in the absence of additional genome sequence data, we have been conservative in our taxonomic assignment.

Two of the most frequently detected phylogroup 1 *pyrH* genotypes corresponded to T. vincentii (pyrH006) and the Treponema sp. IC phylogroup (pyrH004), which is proposed to constitute a distinct oral Treponema species (17). The genome sequence of a reference strain of this proposed new species, Treponema sp. strain OMZ 804 (ATCC 700766), has recently been published (48). It may be noted that the pyrH004 and pyrH006 genotypes were highly prevalent within the gingivitis subjects and were less prevalent in the periodontitis subjects (Fig. 1 and 2), but these differences were not statistically significant (*P = *0.0915 and 0.1534, Fisher’s exact test). However, it is important to stress that the oral cavities of both the periodontitis and gingivitis subjects contained a wide diversity of phylogroup 1 and 2 oral treponeme taxa in their subgingival niches but that there were notable differences in their species compositions.

The diversity of oral treponeme major surface protein (*msp*) genotypes has previously been surveyed in a cohort of subjects with or without periodontitis (45). Consistent with the results presented here, the most frequently detected *msp* genotype (NP2_6) corresponded to the T. denticola ATCC 35405 type strain, which was equally prevalent in both subject groups. However, due to the very high levels of Msp sequence diversity within oral treponemes, it is not possible to directly correlate many of the *pyrH* genotypes described here with the previously defined *msp* genotypes with high levels of confidence.

Our study has several limitations. First, the sample size of our cohort is relatively small. It is large enough to clearly demonstrate that the oral cavities of individuals with periodontitis or gingivitis commonly contain multiple T. denticola lineages (as well as those of other treponeme phylotypes). However, it may not be large enough to indicate how many different clinical strains of the respective species/phylotypes are typically present in an individual subject’s oral cavity. In addition, as we used pooled multisite samples, our genetic analysis presents an overview of phylogroup 1 and 2 treponeme distributions within subgingival plaque biofilm niches in the oral cavities of Hong Kong Chinese individuals. It does not indicate whether multiple lineages of T. denticola or other treponeme species/phylotypes occupy the same clinical site or reside within distinct sites. It should also be noted that the numbers of cloned *pyrH* gene sequences (i.e., clonal abundance) corresponding to each *pyrH* genotype are only semiquantitative indicators of the actual abundance of oral treponeme cells bearing the respective *pyrH* genes on their chromosomes. Thus, the relative abundances of the various treponeme taxa detected in the different subjects (as evaluated by the clonal abundances of the respective *pyrH* genotypes) cannot be directly compared. It should also be noted that, because our study focused exclusively on treponemes belonging to oral phylogroups 1 and 2, the clinical distributions of treponemes belonging to other oral phylogroups were not investigated.

While the *pyrH* gene may be very useful for assigning taxonomy, the precise correlation between *pyrH* gene sequence identity and pangenomic diversity across the many tens of species/species-level phylotypes of (human) oral treponeme taxa remains to be determined. Over forthcoming years, the increased availability of complete genome sequences for diverse Treponema taxa, combined with carefully targeted full-metagenome clinical investigations, will enable the more accurate establishment of their etiological associations with periodontal diseases.

### Conclusions.

Subjects with periodontitis and gingivitis harbor a wide diversity of T. denticola, T. vincentii, and other oral phylogroup 1 and 2 treponeme taxa within subgingival niches. Our results suggest that there may be notable differences in the levels of taxonomic diversity or compositions of treponeme species/phylotypes in subjects with periodontitis versus gingivitis. Notably, individual subjects commonly harbor multiple clinical strains of the same oral treponeme species within their oral cavities.

## MATERIALS AND METHODS

### Subject recruitment.

A cross-sectional design was employed to compare the diversity of oral treponemes in subgingival plaque samples from periodontally diseased sites in subjects presenting with periodontitis and gingivitis. Chinese subjects with a minimum of 20 teeth, excluding third molars and teeth planned for extraction, who attended the Reception Clinic, Prince Philip Dental Hospital, from July 2009 to February 2015 were chosen from the patient pool and were invited to attend a screening session. Smoking history and dental status were extracted from records of first attendance and were confirmed on the screening visit. Trained dentists examined the participants. A calibration exercise on the clinical operations by experienced dentists was carried out prior to the start of the study. The full-mouth %BOP scores, probing pocket depth (PPD), and radiographic bone level (49) were recorded after written informed consent.

Subjects fulfilling the following criteria were invited to partake in the study. The inclusion criteria were as follows: for the periodontitis (P) group, at least two tooth sites with PPD of ≥5 mm in two quadrants (50) with radiographic evidence of interproximal alveolar bone loss of more than 1/3 the root length, and for the gingivitis (G) group, (i) PPD of ≤3 mm on an intact periodontium or PPD of ≤4 mm on a reduced periodontium, (ii) no radiographic bone loss in any standing tooth, and (iii) BOP of ≥10% (51). The exclusion criteria were (i) the presence of conditions suggesting a need for antibiotic prophylaxis prior to periodontal examination and invasive dental treatment, (ii) a history of systemic disease or taking medications known to be associated with periodontal conditions, and (iii) a history of periodontal treatment except oral hygiene instructions or antibiotic therapy in the previous 6 months. The periodontal conditions of P and G group participants were classified according to current recommendations (52).

Ethical approval was granted by the Institutional Review Board of the University of Hong Kong/Hospital Authority Hong Kong Cluster West (UW 15–641), and the study was performed in accordance with the Declaration of Helsinki.

### Sample collection.

Prior to sampling, the target clinical sites were isolated with sterile cotton rolls and air dried. Supragingival plaque was first removed by sterile universal curettes and discarded, and then subgingival plaque samples were collected, using sterilized Gracey curettes, from all the diseased sites (PPD ≥ 5 mm) from the P group participants or from all subgingival sites from the G group participants. Samples from each subject were pooled into a 2-ml, sterile, screw-cap microcentrifuge tube containing 1.0 ml of phosphate-buffered saline (PBS; 0.01 M phosphate, 137 mM NaCl, 2.7 mM KCl, pH 7.4). Samples were stored at −70°C until processing. After thawing, subgingival plaque samples were washed twice with PBS, and genomic DNA was extracted using the QIAamp DNA minikit (Qiagen Group, USA) following the manufacturer’s instructions. DNA was eluted in 100 μl of the manufacturer’s elution buffer.

### PCR amplification and DNA sequencing of treponeme *pyrH* genes.

The *pyrH* gene sequences from phylogroup 1 and 2 treponemes were amplified using the following previously described primer sets (17): for phylogroup 1, pyrH-I-F (ATGGTACGGGTCTTATCGGTAG) and pyrH-I-R (TTAACCTATCGTTGTGCCTTTAA), and for phylogroup 2, pyrH-II-F (ATGGTAACTGTTTTGTCGGT) and pyrH-II-R (TTAGCCGATTACCGTTCCTT). PCRs were carried out on a GeneAmp PCR system 9700 (Applied Biosystems) using a touchdown PCR approach with GoTaq flexi DNA polymerase (1 U; Promega) with conditions as previously described (17). PCR products were gel purified using QIAquick gel extraction kits (Qiagen Group, USA) and TOPO cloned into pCR2.1 TOPO vectors (TOPO TA cloning kit; Invitrogen, Life Technologies, CA, USA). Ligation mixtures were electroporated into Escherichia coli strain DH10B, plated onto Luria-Bertani (LB) 1% agar plates supplemented with kanamycin (50 μg/ml) and X-Gal (5-bromo-4-chloro-indolyl-β-d-galactopyranoside, 20 μg/ml), and then incubated overnight at 37°C. All transformant colonies (*ca*. 26 to 53 for each plate) were inoculated into fresh LB broth plus kanamycin (4.0 ml) and incubated overnight at 37°C, and then plasmid DNA was purified from each sample (QIAprep spin miniprep kits; Qiagen). All plasmid inserts were Sanger sequenced bidirectionally using M13 forward and reverse primers (Beijing Genome Institute [BGI] Hong Kong, Ltd.).

### Bioinformatics/sequence processing and genotype assignment.

The bidirectional Sanger sequences were aligned and primer sequences were trimmed off using BioEdit version 7.2.3 (53). The combined full-length sequences were quality filtered and manually checked. Multiple sequence alignments of gene sequences were constructed using MAFFT version 7.266 (54). Pairwise distance matrices among the aligned DNA sequences were calculated using the *dist.seqs* function in Mothur (55, 56). The *cluster* function was used to group the sequences into OTUs or *pyrH* genotypes (as indicated in the text) by sequence identities based on the average neighbor algorithm. The *pyrH* genotypes were defined using 97% sequence identity cutoffs, based on results from previous analyses (17). Alpha diversity estimators, including genotype richness, diversity, and depth of sample coverage, were calculated using the *summary.single* functions in Mothur.

### Phylogenetic analyses: phylogeny estimation, recombination, and selective pressure detection.

Maximum-likelihood (ML) estimations of phylogenetic trees were calculated for the *pyrH* gene sequence data sets using the program GARLI (Genetic Algorithm for Rapid Likelihood Inference) (57). Prior to the ML estimation, the best substitution model and gamma rate heterogeneity were determined using the Akaike information criterion implemented in jModelTest2 (36). The best ML tree topology with the maximum clade credibility was adopted with clade supports annotated at the branch nodes (58) after 1,000 bootstrapping replicates. Bayesian posterior probabilities were calculated for the best tree topologies using MrBayes version 3 (59). Branch nodes with ≥60% support value were shown in the phylograms.

### Subject- and group-based comparisons of clinical *pyrH* genotype composition.

The absolute abundances of the respective clinical *pyrH* genotypes (i.e., the number of plasmid clones bearing the corresponding *pyrH* sequence) within the participants and subject groups were visualized as heat maps using the R packages superheat and ggplot2 version 2.2.1 (https://www.r-project.org/). Prevalence was defined as the frequency of the clinical *pyrH* genotype being detected within each of the respective subjects. The clinical *pyrH* genotype compositions in each subject were also analyzed using nonmetric multidimensional scaling (nMDS). ML trees containing representative *pyrH* genotypes were used to compute generalized UniFrac distance metrics (60, 61). The results were visualized by 2-dimensional nMDS ordination in R using the phyloseq package (62).

### Statistical tests of demographic and clinical parameters.

Statistical analysis was performed using SPSS software 25 (SPSS, Inc., Chicago, IL, USA) and R package software (3.5.2). The *P* value threshold was set to 0.05. Descriptive statistics were conducted to analyze the clinical parameters. Assumption tests for normality and equality of variance were performed prior to all statistical analyses. Standard errors were calculated to estimate the sampling errors on age, full-mouth BOP scores (%), full-mouth PPD of ≥5 mm (%), number of sites sampled, and mean PPD for sampled sites. The interquartile ranges were calculated to estimate the sampling errors on standing teeth, %BOP scores for sampled sites, numbers of cloned *pyrH* sequences, and numbers of *pyrH* genotypes. Gender differences between the two clinical groups were compared using Fisher’s exact test. The independent-samples *t* test was employed to compare age, full-mouth %BOP scores, and the number of sites sampled between the P and G groups. The one-sample *t* test was used to compare the mean PPD values for sampled sites. The Mann-Whitney U test was used to compare the numbers of standing teeth, %BOP scores for sampled sites, numbers of cloned *pyrH* sequences, and numbers of *pyrH* genotypes between the P and G groups.

### Data availability.

All full-length cloned *pyrH* sequences were deposited in NCBI GenBank with accession numbers MT091982 to MT093184 (Table S5).
