# Supplementary material for: Diversity of Treponema denticola and Other Oral Treponeme Lineages in Subjects with Periodontitis and Gingivitis
Source: Microbiol Spectr. 2021 Sep 29;9(2):e00701-21. doi: 10.1128/Spectrum.00701-21 (PMC8557910; doi:10.1128/Spectrum.00701-21)
Supplement: SUPPLEMENTAL FILE 1 — Supplemental material. Download SPECTRUM00701-21_Supp_1_seq9.pdf, PDF file, 0.5 MB [file spectrum00701-21_supp_1_seq9.pdf]

SUPPLEMENTARY MATERIAL

Diversity of *Treponema denticola* and other oral treponeme lineages in subjects with periodontitis and gingivitis

Huihui Zeng[a+], Yuki Chan[a+], Wenling Gao[a,b], W. Keung Leung[a], Rory M. Watt[a*]

[a] Faculty of Dentistry, The University of Hong Kong, Hong Kong SAR, China

[b] Current affiliation: Guanghua School of Stomatology, Hospital of Stomatology, Guangdong Provincial Key Laboratory of Stomatology, Sun Yat-sen University, Guangzhou, PR China

+These authors contributed equally

Correspondence to: Dr. Rory M. Watt: rmwatt@hku.hk

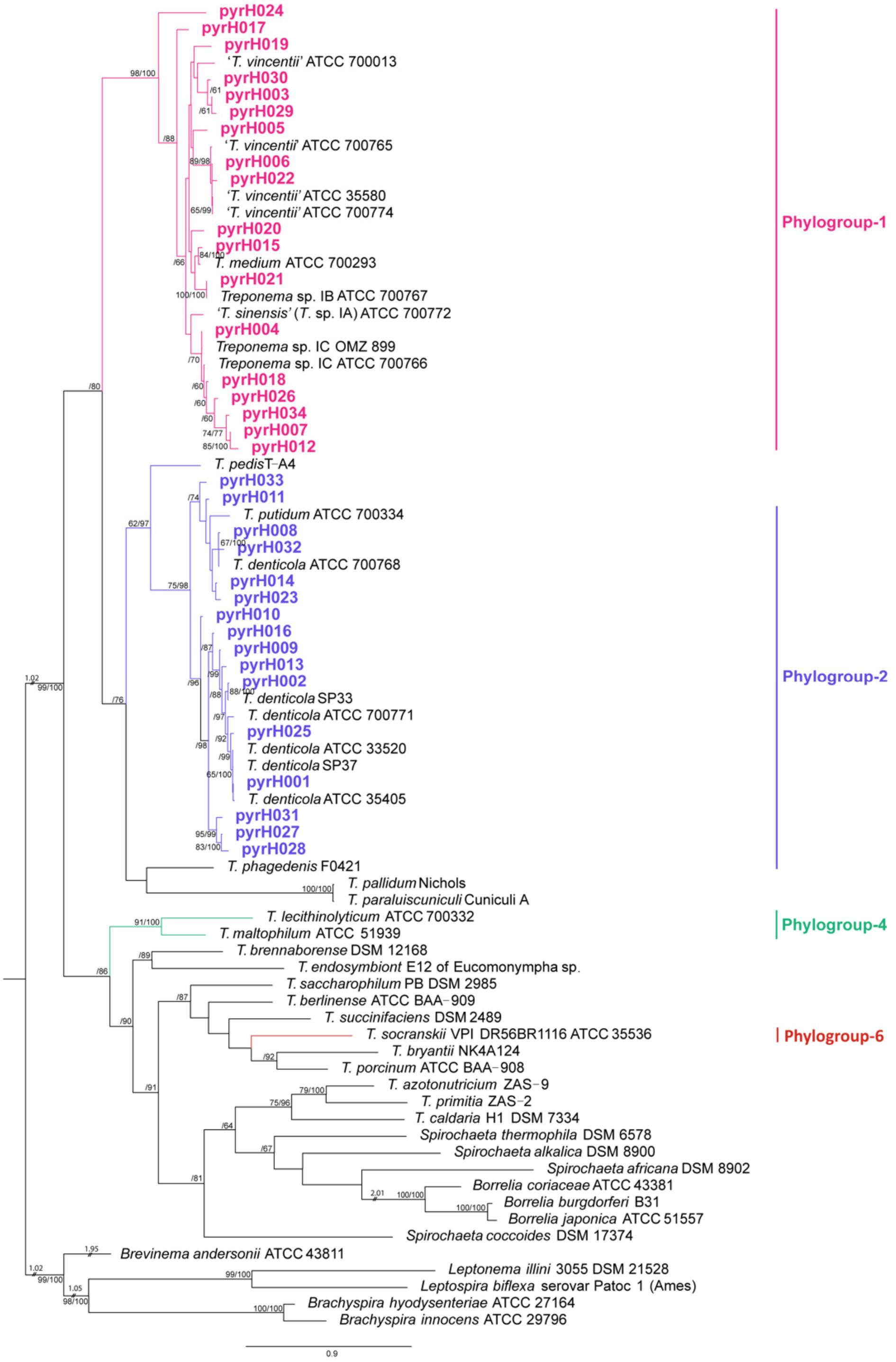

Figure S1 Maximum Likelihood phylogenetic tree of treponeme *pyrH* genes. The 34 *pyrH* genotypes identified in this study are indicated with bold-text. The pink and purple font colors indicate genotypes belonging to oral phylogroup 1 and 2, respectively. A scale bar indicates 0.9 nucleotide changes per position. Other explanatory details refer to Fig. 1.

Table S1 Number of cloned *pyrH* gene sequences and genotypes obtained within each subject.

Subject no.	Number of *pyrH* sequences obtained			Number of *pyrH* genotypes identified		
	**Total**	**Phylogroup-1 treponemes**	**Phylogroup-2 treponemes**	**Total**	**Phylogroup-1 treponemes**	**Phylogroup-2 treponemes**
P1	59	29	30	8	5	3
P2	63	30	33	11	6	5
P3	66	30	36	12	6	6
P4	60	30	30	4	2	2
P5	60	30	30	5	3	2
P6	60	30	30	6	3	3
P7	76	30	46	6	4	2
P8	80	30	50	5	3	2
P9	81	29	52	8	2	6
P10	59	29	30	8	4	4
G1	79	30	49	4	3	1
G2	83	30	53	5	3	2
G3	81	30	51	5	3	2
G4	63	30	33	5	3	2
G5	59	30	29	4	3	1
G6	60	30	30	5	4	1
G7	60	30	30	7	4	3
G8	55	29	26	5	4	1
**Total**	1,204	536	668	34	18	16

Phylogroup-1 treponemes: Number of treponeme phylogroup 1 *pyrH* gene sequences or genotypes. **Phylogroup-2 treponemes:** Number of treponeme phylogroup 2 *pyrH* gene sequences or genotypes. **Total:** Total number of *pyrH* gene sequences or genotypes.

(Phylogroup 1) in (P): Name of treponeme phylogroup 1 *pyrH* genes identified in periodontitis (P) subjects. **(Phylogroup 1) in (G)**: Name of treponeme phylogroup 1 *pyrH* genes identified in gingivitis (G) subjects. **(Phylogroup 2) in (P)**: Name of treponeme phylogroup 2 *pyrH* genes identified in periodontitis (P) subjects. **(Phylogroup 2) in (G)**: Name of treponeme phylogroup 2 *pyrH* genes identified in periodontitis (G) subjects. The identity of the clone is indicated, e.g. Grp1_P1_1 refers to the first clone of oral treponeme phylogroup 1 *pyrH* gene sequence from patient P1.

Table S2. Summary of taxonomy and distributions of oral treponeme *pyrH* genotypes and cloned *pyrH* gene sequences identified in P and G subject groups.

***pyrH* genotype**	**(P)**	**(G)**	**(P+G)**	**Oral treponeme phylogroup**	**Species / phylotype**
pyrH001	135	191	326	2	*T. denticola*
pyrH002	96	99	195	2	*T. denticola*
pyrH003	118	51	169	1	*Treponema* sp. I*
pyrH004	15	81	96	1	*Treponema* sp. IC
pyrH005	66	24	90	1	*Treponema* sp. I*
pyrH006	7	58	65	1	*'T. vincentii'*
pyrH007	53	15	68	1	*Treponema* sp. I*
pyrH008	28	3	31	2	'atypical' *T. denticola*
pyrH009	32	0	32	2	*Treponema* sp. II*
pyrH010	26	0	26	2	*Treponema* sp. II*
pyrH011	18	7	25	2	*Treponema* sp. II*
pyrH012	20	0	20	1	*Treponema* sp. I*
pyrH013	10	0	10	2	*T. denticola*
pyrH014	10	0	10	2	*Treponema* sp. II*
pyrH015	8	0	8	1	*T. medium*
pyrH016	6	0	6	2	*Treponema* sp. II*
pyrH017	4	0	4	1	*Treponema* sp. I*
pyrH018	0	3	3	1	*Treponema* sp. I*
pyrH019	0	3	3	1	*Treponema* sp. I*
pyrH020	2	0	2	1	*Treponema* sp. I*
pyrH021	2	0	2	1	*Treponema* sp. IB
pyrH022	0	1	1	1	*Treponema* sp. I*
pyrH023	1	0	1	2	*Treponema* sp. II*
pyrH024	1	0	1	1	*Treponema* sp. I*
pyrH025	1	0	1	2	*T. denticola*
pyrH026	0	1	1	1	*Treponema* sp. I*
pyrH027	1	0	1	2	*Treponema* sp. II*
pyrH028	1	0	1	2	*Treponema* sp. II*
pyrH029	1	0	1	1	*Treponema* sp. I*
pyrH030	0	1	1	1	*Treponema* sp. I*
pyrH031	1	0	1	2	*Treponema* sp. II*
pyrH032	0	1	1	2	'atypical' *T. denticola*
pyrH033	1	0	1	2	*Treponema* sp. II*
pyrH034	0	1	1	1	*Treponema* sp. I*

(P) Number of cloned *pyrH* gene sequences corresponding to this *pyrH* genotype obtained from periodontitis (P) subjects. **(G)** Number of cloned *pyrH* gene sequences corresponding to this *pyrH* genotype obtained from gingivitis (G) subjects. **(P+G)** Total number of cloned *pyrH* gene sequences obtained that corresponded to this *pyrH* genotype. Taxonomy is assigned with reference to the sequences described in [Chen T, Yu WH, Izard J, Baranova OV, Lakshmanan A, Dewhirst FE. 2010. The Human Oral Microbiome Database: a web accessible resource for investigating oral microbe taxonomic and genomic information. *Database* (Oxford) 2010:baq013]. * Indicates that the corresponding oral treponeme species/phylotype cannot be assigned with confidence.

Table S3. Alpha diversity indices for oral treponeme phylogroup 1 *pyrH* genotypes detected within each subject.

Subject	nseqs	Good's coverage	sobs	Simpson	Chao1	Shannon	ACE
P1	29	0.97	5.00	0.37	5.00	1.21	5.36
P2	30	0.90	6.00	0.30	9.00	1.34	18.08
P3	30	0.93	6.00	0.49	6.33	1.07	7.43
P4	30	1.00	2.00	0.49	2.00	0.69	0.00
P5	30	0.97	3.00	0.62	3.00	0.64	4.76
P6	30	0.97	3.00	0.50	3.00	0.77	4.87
P7	30	0.97	4.00	0.39	4.00	1.01	5.00
P8	30	1.00	3.00	0.49	3.00	0.86	3.00
P9	29	1.00	2.00	0.87	2.00	0.25	2.00
P10	29	1.00	4.00	0.31	4.00	1.23	4.00
G1	30	0.97	2.00	0.45	3.00	0.82	0.00
G2	30	1.00	3.00	0.65	3.00	0.63	3.00
G3	30	1.00	3.00	0.49	3.00	0.86	3.00
G4	30	1.00	4.00	0.44	3.00	0.90	3.00
G5	30	0.97	3.00	0.58	3.00	0.68	4.80
G6	30	0.97	3.00	0.64	4.00	0.70	4.60
G7	30	0.93	4.00	0.43	5.00	0.93	0.00
G8	29	0.97	4.00	0.36	4.00	1.09	4.76

Nseq is the number of sequences observed; **Good's coverage** calculates Good's coverage values; **sobs** is the number of detected genotypes; **Simpson** is the Simpson diversity index; **Chao1** represents the Chao1 richness index; **Shannon** represents the Shannon diversity index; **ACE** is the abundance-based coverage ACE richness estimator.

Table S4. Alpha diversity indices for oral treponeme phylogroup 2 *pyrH* genotypes detected within periodontitis and gingivitis subjects.

Subject	nseqs	Good's coverage	sobs	Simpson	Chao1	Shannon	ACE
P1	30	0.97	3.00	0.75	3.00	0.47	4.11
P2	33	0.94	5.00	0.41	6.00	1.10	7.23
P3	36	0.94	6.00	0.23	7.00	1.50	7.60
P4	30	1.00	2.00	0.49	2.00	0.68	0.00
P5	30	1.00	2.00	0.63	2.00	0.54	2.00
P6	30	1.00	3.00	0.42	3.00	0.89	3.00
P7	46	0.98	2.00	0.96	2.00	0.10	0.00
P8	50	0.98	2.00	0.96	2.00	0.10	0.00
P9	52	0.96	6.00	0.39	7.00	1.21	7.63
P10	30	0.97	4.00	0.29	4.00	1.20	4.54
G1	49	1.00	1.00	1.00	1.00	0.00	0.00
G2	53	1.00	2.00	0.74	2.00	0.42	2.00
G3	51	1.00	2.00	0.92	2.00	0.17	2.00
G4	33	1.00	2.00	0.66	2.00	0.52	2.00
G5	29	1.00	1.00	1.00	1.00	0.00	0.00
G6	30	1.00	1.00	1.00	1.00	0.00	0.00
G7	30	0.97	3.00	0.75	3.00	0.47	4.11
G8	26	1.00	1.00	1.00	1.00	0.00	0.00

Explanatory details refer to Table S3.

Table S5. NCBI GenBank accession numbers for cloned *pyrH* gene sequences identified in this study.

(Phylogroup 1) in (P)		(Phylogroup 1) in (G)		(Phylogroup 2) in (P)		(Phylogroup 2) in (G)	
Clone	Accession	Clone	Accession	Clone	Accession	Clone	Accession
Grp1_P1_1	MT091982	Grp1_G1_1	MT092279	Grp2_P1_1	MT092518	Grp2_G1_1	MT092885
Grp1_P1_2	MT091983	Grp1_G1_2	MT092280	Grp2_P1_2	MT092519	Grp2_G1_2	MT092886
Grp1_P1_3	MT091984	Grp1_G1_3	MT092281	Grp2_P1_3	MT092520	Grp2_G1_3	MT092887
Grp1_P1_4	MT091985	Grp1_G1_4	MT092282	Grp2_P1_4	MT092521	Grp2_G1_4	MT092888
Grp1_P1_5	MT091986	Grp1_G1_5	MT092283	Grp2_P1_5	MT092522	Grp2_G1_5	MT092889
Grp1_P1_6	MT091987	Grp1_G1_6	MT092284	Grp2_P1_6	MT092523	Grp2_G1_6	MT092890
Grp1_P1_7	MT091988	Grp1_G1_7	MT092285	Grp2_P1_7	MT092524	Grp2_G1_7	MT092891
Grp1_P1_8	MT091989	Grp1_G1_8	MT092286	Grp2_P1_8	MT092525	Grp2_G1_8	MT092892
Grp1_P1_9	MT091990	Grp1_G1_9	MT092287	Grp2_P1_9	MT092526	Grp2_G1_9	MT092893
Grp1_P1_10	MT091991	Grp1_G1_10	MT092288	Grp2_P1_10	MT092527	Grp2_G1_10	MT092894
Grp1_P1_11	MT091992	Grp1_G1_11	MT092289	Grp2_P1_11	MT092528	Grp2_G1_11	MT092895
Grp1_P1_12	MT091993	Grp1_G1_12	MT092290	Grp2_P1_12	MT092529	Grp2_G1_12	MT092896
Grp1_P1_13	MT091994	Grp1_G1_13	MT092291	Grp2_P1_13	MT092530	Grp2_G1_13	MT092897
Grp1_P1_14	MT091995	Grp1_G1_14	MT092292	Grp2_P1_14	MT092531	Grp2_G1_14	MT092898
Grp1_P1_15	MT091996	Grp1_G1_15	MT092293	Grp2_P1_15	MT092532	Grp2_G1_15	MT092899
Grp1_P1_16	MT091997	Grp1_G1_16	MT092294	Grp2_P1_16	MT092533	Grp2_G1_16	MT092900
Grp1_P1_17	MT091998	Grp1_G1_17	MT092295	Grp2_P1_17	MT092534	Grp2_G1_17	MT092901
Grp1_P1_18	MT091999	Grp1_G1_18	MT092296	Grp2_P1_18	MT092535	Grp2_G1_18	MT092902
Grp1_P1_19	MT092000	Grp1_G1_19	MT092297	Grp2_P1_19	MT092536	Grp2_G1_19	MT092903
Grp1_P1_20	MT092001	Grp1_G1_20	MT092298	Grp2_P1_20	MT092537	Grp2_G1_20	MT092904
Grp1_P1_21	MT092002	Grp1_G1_21	MT092299	Grp2_P1_21	MT092538	Grp2_G1_21	MT092905
Grp1_P1_22	MT092003	Grp1_G1_22	MT092300	Grp2_P1_22	MT092539	Grp2_G1_22	MT092906
Grp1_P1_23	MT092004	Grp1_G1_23	MT092301	Grp2_P1_23	MT092540	Grp2_G1_23	MT092907
Grp1_P1_24	MT092005	Grp1_G1_24	MT092302	Grp2_P1_24	MT092541	Grp2_G1_24	MT092908
Grp1_P1_25	MT092006	Grp1_G1_25	MT092303	Grp2_P1_25	MT092542	Grp2_G1_25	MT092909
Grp1_P1_26	MT092007	Grp1_G1_26	MT092304	Grp2_P1_26	MT092543	Grp2_G1_26	MT092910
Grp1_P1_27	MT092008	Grp1_G1_27	MT092305	Grp2_P1_27	MT092544	Grp2_G1_27	MT092911
Grp1_P1_28	MT092009	Grp1_G1_28	MT092306	Grp2_P1_28	MT092545	Grp2_G1_28	MT092912
Grp1_P1_29	MT092010	Grp1_G1_29	MT092307	Grp2_P1_29	MT092546	Grp2_G1_29	MT092913
Grp1_P4_1	MT092011	Grp1_G1_30	MT092308	Grp2_P1_30	MT092547	Grp2_G1_30	MT092914
Grp1_P4_2	MT092012	Grp1_G2_1	MT092309	Grp2_P4_1	MT092548	Grp2_G1_31	MT092915
Grp1_P4_3	MT092013	Grp1_G2_2	MT092310	Grp2_P4_2	MT092549	Grp2_G1_32	MT092916
Grp1_P4_4	MT092014	Grp1_G2_3	MT092311	Grp2_P4_3	MT092550	Grp2_G1_33	MT092917
Grp1_P4_5	MT092015	Grp1_G2_4	MT092312	Grp2_P4_4	MT092551	Grp2_G1_34	MT092918
Grp1_P4_6	MT092016	Grp1_G2_5	MT092313	Grp2_P4_5	MT092552	Grp2_G1_35	MT092919
Grp1_P4_7	MT092017	Grp1_G2_6	MT092314	Grp2_P4_6	MT092553	Grp2_G1_36	MT092920
Grp1_P4_8	MT092018	Grp1_G2_7	MT092315	Grp2_P4_7	MT092554	Grp2_G1_37	MT092921
Grp1_P4_9	MT092019	Grp1_G2_8	MT092316	Grp2_P4_8	MT092555	Grp2_G1_38	MT092922
Grp1_P4_10	MT092020	Grp1_G2_9	MT092317	Grp2_P4_9	MT092556	Grp2_G1_39	MT092923
Grp1_P4_11	MT092021	Grp1_G2_10	MT092318	Grp2_P4_10	MT092557	Grp2_G1_40	MT092924
Grp1_P4_12	MT092022	Grp1_G2_11	MT092319	Grp2_P4_11	MT092558	Grp2_G1_41	MT092925
Grp1_P4_13	MT092023	Grp1_G2_12	MT092320	Grp2_P4_12	MT092559	Grp2_G1_42	MT092926
Grp1_P4_14	MT092024	Grp1_G2_13	MT092321	Grp2_P4_13	MT092560	Grp2_G1_43	MT092927
Grp1_P4_15	MT092025	Grp1_G2_14	MT092322	Grp2_P4_14	MT092561	Grp2_G1_44	MT092928
Grp1_P4_16	MT092026	Grp1_G2_15	MT092323	Grp2_P4_15	MT092562	Grp2_G1_45	MT092929
Grp1_P4_17	MT092027	Grp1_G2_16	MT092324	Grp2_P4_16	MT092563	Grp2_G1_46	MT092930
Grp1_P4_18	MT092028	Grp1_G2_17	MT092325	Grp2_P4_17	MT092564	Grp2_G1_47	MT092931
Grp1_P4_19	MT092029	Grp1_G2_18	MT092326	Grp2_P4_18	MT092565	Grp2_G1_48	MT092932
Grp1_P4_20	MT092030	Grp1_G2_19	MT092327	Grp2_P4_19	MT092566	Grp2_G1_49	MT092933
Grp1_P4_21	MT092031	Grp1_G2_20	MT092328	Grp2_P4_20	MT092567	Grp2_G2_1	MT092934
Grp1_P4_22	MT092032	Grp1_G2_21	MT092329	Grp2_P4_21	MT092568	Grp2_G2_2	MT092935
Grp1_P4_23	MT092033	Grp1_G2_22	MT092330	Grp2_P4_22	MT092569	Grp2_G2_3	MT092936

(Phylogroup 1) in (P)		(Phylogroup 1) in (G)		(Phylogroup 2) in (P)		(Phylogroup 2) in (G)	
Clone	Accession	Clone	Accession	Clone	Accession	Clone	Accession
Grp1_P4_24	MT092034	Grp1_G2_23	MT092331	Grp2_P4_23	MT092570	Grp2_G2_4	MT092937
Grp1_P4_25	MT092035	Grp1_G2_24	MT092332	Grp2_P4_24	MT092571	Grp2_G2_5	MT092938
Grp1_P4_26	MT092036	Grp1_G2_25	MT092333	Grp2_P4_25	MT092572	Grp2_G2_6	MT092939
Grp1_P4_27	MT092037	Grp1_G2_26	MT092334	Grp2_P4_26	MT092573	Grp2_G2_7	MT092940
Grp1_P4_28	MT092038	Grp1_G2_27	MT092335	Grp2_P4_27	MT092574	Grp2_G2_8	MT092941
Grp1_P4_29	MT092039	Grp1_G2_28	MT092336	Grp2_P4_28	MT092575	Grp2_G2_9	MT092942
Grp1_P4_30	MT092040	Grp1_G2_29	MT092337	Grp2_P4_29	MT092576	Grp2_G2_10	MT092943
Grp1_P5_1	MT092041	Grp1_G2_30	MT092338	Grp2_P4_30	MT092577	Grp2_G2_11	MT092944
Grp1_P5_2	MT092042	Grp1_G3_1	MT092339	Grp2_P5_1	MT092578	Grp2_G2_12	MT092945
Grp1_P5_3	MT092043	Grp1_G3_2	MT092340	Grp2_P5_2	MT092579	Grp2_G2_13	MT092946
Grp1_P5_4	MT092044	Grp1_G3_3	MT092341	Grp2_P5_3	MT092580	Grp2_G2_14	MT092947
Grp1_P5_5	MT092045	Grp1_G3_4	MT092342	Grp2_P5_4	MT092581	Grp2_G2_15	MT092948
Grp1_P5_6	MT092046	Grp1_G3_5	MT092343	Grp2_P5_5	MT092582	Grp2_G2_16	MT092949
Grp1_P5_7	MT092047	Grp1_G3_6	MT092344	Grp2_P5_6	MT092583	Grp2_G2_17	MT092950
Grp1_P5_8	MT092048	Grp1_G3_7	MT092345	Grp2_P5_7	MT092584	Grp2_G2_18	MT092951
Grp1_P5_9	MT092049	Grp1_G3_8	MT092346	Grp2_P5_8	MT092585	Grp2_G2_19	MT092952
Grp1_P5_10	MT092050	Grp1_G3_9	MT092347	Grp2_P5_9	MT092586	Grp2_G2_20	MT092953
Grp1_P5_11	MT092051	Grp1_G3_10	MT092348	Grp2_P5_10	MT092587	Grp2_G2_21	MT092954
Grp1_P5_12	MT092052	Grp1_G3_11	MT092349	Grp2_P5_11	MT092588	Grp2_G2_22	MT092955
Grp1_P5_13	MT092053	Grp1_G3_12	MT092350	Grp2_P5_12	MT092589	Grp2_G2_23	MT092956
Grp1_P5_14	MT092054	Grp1_G3_13	MT092351	Grp2_P5_13	MT092590	Grp2_G2_24	MT092957
Grp1_P5_15	MT092055	Grp1_G3_14	MT092352	Grp2_P5_14	MT092591	Grp2_G2_25	MT092958
Grp1_P5_16	MT092056	Grp1_G3_15	MT092353	Grp2_P5_15	MT092592	Grp2_G2_26	MT092959
Grp1_P5_17	MT092057	Grp1_G3_16	MT092354	Grp2_P5_16	MT092593	Grp2_G2_27	MT092960
Grp1_P5_18	MT092058	Grp1_G3_17	MT092355	Grp2_P5_17	MT092594	Grp2_G2_28	MT092961
Grp1_P5_19	MT092059	Grp1_G3_18	MT092356	Grp2_P5_18	MT092595	Grp2_G2_29	MT092962
Grp1_P5_20	MT092060	Grp1_G3_19	MT092357	Grp2_P5_19	MT092596	Grp2_G2_30	MT092963
Grp1_P5_21	MT092061	Grp1_G3_20	MT092358	Grp2_P5_20	MT092597	Grp2_G2_31	MT092964
Grp1_P5_22	MT092062	Grp1_G3_21	MT092359	Grp2_P5_21	MT092598	Grp2_G2_32	MT092965
Grp1_P5_23	MT092063	Grp1_G3_22	MT092360	Grp2_P5_22	MT092599	Grp2_G2_33	MT092966
Grp1_P5_24	MT092064	Grp1_G3_23	MT092361	Grp2_P5_23	MT092600	Grp2_G2_34	MT092967
Grp1_P5_25	MT092065	Grp1_G3_24	MT092362	Grp2_P5_24	MT092601	Grp2_G2_35	MT092968
Grp1_P5_26	MT092066	Grp1_G3_25	MT092363	Grp2_P5_25	MT092602	Grp2_G2_36	MT092969
Grp1_P5_27	MT092067	Grp1_G3_26	MT092364	Grp2_P5_26	MT092603	Grp2_G2_37	MT092970
Grp1_P5_28	MT092068	Grp1_G3_27	MT092365	Grp2_P5_27	MT092604	Grp2_G2_38	MT092971
Grp1_P5_29	MT092069	Grp1_G3_28	MT092366	Grp2_P5_28	MT092605	Grp2_G2_39	MT092972
Grp1_P5_30	MT092070	Grp1_G3_29	MT092367	Grp2_P5_29	MT092606	Grp2_G2_40	MT092973
Grp1_P6_1	MT092071	Grp1_G3_30	MT092368	Grp2_P5_30	MT092607	Grp2_G2_41	MT092974
Grp1_P6_2	MT092072	Grp1_G4_1	MT092369	Grp2_P6_1	MT092608	Grp2_G2_42	MT092975
Grp1_P6_3	MT092073	Grp1_G4_2	MT092370	Grp2_P6_2	MT092609	Grp2_G2_43	MT092976
Grp1_P6_4	MT092074	Grp1_G4_3	MT092371	Grp2_P6_3	MT092610	Grp2_G2_44	MT092977
Grp1_P6_5	MT092075	Grp1_G4_4	MT092372	Grp2_P6_4	MT092611	Grp2_G2_45	MT092978
Grp1_P6_6	MT092076	Grp1_G4_5	MT092373	Grp2_P6_5	MT092612	Grp2_G2_46	MT092979
Grp1_P6_7	MT092077	Grp1_G4_6	MT092374	Grp2_P6_6	MT092613	Grp2_G2_47	MT092980
Grp1_P6_8	MT092078	Grp1_G4_7	MT092375	Grp2_P6_7	MT092614	Grp2_G2_48	MT092981
Grp1_P6_9	MT092079	Grp1_G4_8	MT092376	Grp2_P6_8	MT092615	Grp2_G2_49	MT092982
Grp1_P6_10	MT092080	Grp1_G4_9	MT092377	Grp2_P6_9	MT092616	Grp2_G2_50	MT092983
Grp1_P6_11	MT092081	Grp1_G4_10	MT092378	Grp2_P6_10	MT092617	Grp2_G2_51	MT092984
Grp1_P6_12	MT092082	Grp1_G4_11	MT092379	Grp2_P6_11	MT092618	Grp2_G2_52	MT092985
Grp1_P6_13	MT092083	Grp1_G4_12	MT092380	Grp2_P6_12	MT092619	Grp2_G2_53	MT092986
Grp1_P6_14	MT092084	Grp1_G4_13	MT092381	Grp2_P6_13	MT092620	Grp2_G3_1	MT092987
Grp1_P6_15	MT092085	Grp1_G4_14	MT092382	Grp2_P6_14	MT092621	Grp2_G3_2	MT092988
Grp1_P6_16	MT092086	Grp1_G4_15	MT092383	Grp2_P6_15	MT092622	Grp2_G3_3	MT092989
Grp1_P6_17	MT092087	Grp1_G4_16	MT092384	Grp2_P6_16	MT092623	Grp2_G3_4	MT092990
Grp1_P6_18	MT092088	Grp1_G4_17	MT092385	Grp2_P6_17	MT092624	Grp2_G3_5	MT092991
Grp1_P6_19	MT092089	Grp1_G4_18	MT092386	Grp2_P6_18	MT092625	Grp2_G3_6	MT092992
Grp1_P6_20	MT092090	Grp1_G4_19	MT092387	Grp2_P6_19	MT092626	Grp2_G3_7	MT092993
Grp1_P6_21	MT092091	Grp1_G4_20	MT092388	Grp2_P6_20	MT092627	Grp2_G3_8	MT092994
Grp1_P6_22	MT092092	Grp1_G4_21	MT092389	Grp2_P6_21	MT092628	Grp2_G3_9	MT092995
Grp1_P6_23	MT092093	Grp1_G4_22	MT092390	Grp2_P6_22	MT092629	Grp2_G3_10	MT092996

(Phylogroup 1) in (P)		(Phylogroup 1) in (G)		(Phylogroup 2) in (P)		(Phylogroup 2) in (G)	
**Clone**	**Accession**	**Clone**	**Accession**	**Clone**	**Accession**	**Clone**	**Accession**
Grp1_P6_24	MT092094	Grp1_G4_23	MT092391	Grp2_P6_23	MT092630	Grp2_G3_11	MT092997
Grp1_P6_25	MT092095	Grp1_G4_24	MT092392	Grp2_P6_24	MT092631	Grp2_G3_12	MT092998
Grp1_P6_26	MT092096	Grp1_G4_25	MT092393	Grp2_P6_25	MT092632	Grp2_G3_13	MT092999
Grp1_P6_27	MT092097	Grp1_G4_26	MT092394	Grp2_P6_26	MT092633	Grp2_G3_14	MT093000
Grp1_P6_28	MT092098	Grp1_G4_27	MT092395	Grp2_P6_27	MT092634	Grp2_G3_15	MT093001
Grp1_P6_29	MT092099	Grp1_G4_28	MT092396	Grp2_P6_28	MT092635	Grp2_G3_16	MT093002
Grp1_P6_30	MT092100	Grp1_G4_29	MT092397	Grp2_P6_29	MT092636	Grp2_G3_17	MT093003
Grp1_P10_1	MT092101	Grp1_G4_30	MT092398	Grp2_P6_30	MT092637	Grp2_G3_18	MT093004
Grp1_P10_2	MT092102	Grp1_G5_1	MT092399	Grp2_P10_1	MT092638	Grp2_G3_19	MT093005
Grp1_P10_3	MT092103	Grp1_G5_2	MT092400	Grp2_P10_2	MT092639	Grp2_G3_20	MT093006
Grp1_P10_4	MT092104	Grp1_G5_3	MT092401	Grp2_P10_3	MT092640	Grp2_G3_21	MT093007
Grp1_P10_5	MT092105	Grp1_G5_4	MT092402	Grp2_P10_4	MT092641	Grp2_G3_22	MT093008
Grp1_P10_6	MT092106	Grp1_G5_5	MT092403	Grp2_P10_5	MT092642	Grp2_G3_23	MT093009
Grp1_P10_7	MT092107	Grp1_G5_6	MT092404	Grp2_P10_6	MT092643	Grp2_G3_24	MT093010
Grp1_P10_8	MT092108	Grp1_G5_7	MT092405	Grp2_P10_7	MT092644	Grp2_G3_25	MT093011
Grp1_P10_9	MT092109	Grp1_G5_8	MT092406	Grp2_P10_8	MT092645	Grp2_G3_26	MT093012
Grp1_P10_10	MT092110	Grp1_G5_9	MT092407	Grp2_P10_9	MT092646	Grp2_G3_27	MT093013
Grp1_P10_11	MT092111	Grp1_G5_10	MT092408	Grp2_P10_10	MT092647	Grp2_G3_28	MT093014
Grp1_P10_12	MT092112	Grp1_G5_11	MT092409	Grp2_P10_11	MT092648	Grp2_G3_29	MT093015
Grp1_P10_13	MT092113	Grp1_G5_12	MT092410	Grp2_P10_12	MT092649	Grp2_G3_30	MT093016
Grp1_P10_14	MT092114	Grp1_G5_13	MT092411	Grp2_P10_13	MT092650	Grp2_G3_31	MT093017
Grp1_P10_15	MT092115	Grp1_G5_14	MT092412	Grp2_P10_14	MT092651	Grp2_G3_32	MT093018
Grp1_P10_16	MT092116	Grp1_G5_15	MT092413	Grp2_P10_15	MT092652	Grp2_G3_33	MT093019
Grp1_P10_17	MT092117	Grp1_G5_16	MT092414	Grp2_P10_16	MT092653	Grp2_G3_34	MT093020
Grp1_P10_18	MT092118	Grp1_G5_17	MT092415	Grp2_P10_17	MT092654	Grp2_G3_35	MT093021
Grp1_P10_19	MT092119	Grp1_G5_18	MT092416	Grp2_P10_18	MT092655	Grp2_G3_36	MT093022
Grp1_P10_20	MT092120	Grp1_G5_19	MT092417	Grp2_P10_19	MT092656	Grp2_G3_37	MT093023
Grp1_P10_21	MT092121	Grp1_G5_20	MT092418	Grp2_P10_20	MT092657	Grp2_G3_38	MT093024
Grp1_P10_22	MT092122	Grp1_G5_21	MT092419	Grp2_P10_21	MT092658	Grp2_G3_39	MT093025
Grp1_P10_23	MT092123	Grp1_G5_22	MT092420	Grp2_P10_22	MT092659	Grp2_G3_40	MT093026
Grp1_P10_24	MT092124	Grp1_G5_23	MT092421	Grp2_P10_23	MT092660	Grp2_G3_41	MT093027
Grp1_P10_25	MT092125	Grp1_G5_24	MT092422	Grp2_P10_24	MT092661	Grp2_G3_42	MT093028
Grp1_P10_26	MT092126	Grp1_G5_25	MT092423	Grp2_P10_25	MT092662	Grp2_G3_43	MT093029
Grp1_P10_27	MT092127	Grp1_G5_26	MT092424	Grp2_P10_26	MT092663	Grp2_G3_44	MT093030
Grp1_P10_28	MT092128	Grp1_G5_27	MT092425	Grp2_P10_27	MT092664	Grp2_G3_45	MT093031
Grp1_P10_29	MT092129	Grp1_G5_28	MT092426	Grp2_P10_28	MT092665	Grp2_G3_46	MT093032
Grp1_P7_1	MT092130	Grp1_G5_29	MT092427	Grp2_P10_29	MT092666	Grp2_G3_47	MT093033
Grp1_P7_2	MT092131	Grp1_G5_30	MT092428	Grp2_P10_30	MT092667	Grp2_G3_48	MT093034
Grp1_P7_3	MT092132	Grp1_G6_1	MT092429	Grp2_P7_1	MT092668	Grp2_G3_49	MT093035
Grp1_P7_4	MT092133	Grp1_G6_2	MT092430	Grp2_P7_2	MT092669	Grp2_G3_50	MT093036
Grp1_P7_5	MT092134	Grp1_G6_3	MT092431	Grp2_P7_3	MT092670	Grp2_G3_51	MT093037
Grp1_P7_6	MT092135	Grp1_G6_4	MT092432	Grp2_P7_4	MT092671	Grp2_G4_1	MT093038
Grp1_P7_7	MT092136	Grp1_G6_5	MT092433	Grp2_P7_5	MT092672	Grp2_G4_2	MT093039
Grp1_P7_8	MT092137	Grp1_G6_6	MT092434	Grp2_P7_6	MT092673	Grp2_G4_3	MT093040
Grp1_P7_9	MT092138	Grp1_G6_7	MT092435	Grp2_P7_7	MT092674	Grp2_G4_4	MT093041
Grp1_P7_10	MT092139	Grp1_G6_8	MT092436	Grp2_P7_8	MT092675	Grp2_G4_5	MT093042
Grp1_P7_11	MT092140	Grp1_G6_9	MT092437	Grp2_P7_9	MT092676	Grp2_G4_6	MT093043
Grp1_P7_12	MT092141	Grp1_G6_10	MT092438	Grp2_P7_10	MT092677	Grp2_G4_7	MT093044
Grp1_P7_13	MT092142	Grp1_G6_11	MT092439	Grp2_P7_11	MT092678	Grp2_G4_8	MT093045
Grp1_P7_14	MT092143	Grp1_G6_12	MT092440	Grp2_P7_12	MT092679	Grp2_G4_9	MT093046
Grp1_P7_15	MT092144	Grp1_G6_13	MT092441	Grp2_P7_13	MT092680	Grp2_G4_10	MT093047
Grp1_P7_16	MT092145	Grp1_G6_14	MT092442	Grp2_P7_14	MT092681	Grp2_G4_11	MT093048
Grp1_P7_17	MT092146	Grp1_G6_15	MT092443	Grp2_P7_15	MT092682	Grp2_G4_12	MT093049
Grp1_P7_18	MT092147	Grp1_G6_16	MT092444	Grp2_P7_16	MT092683	Grp2_G4_13	MT093050
Grp1_P7_19	MT092148	Grp1_G6_17	MT092445	Grp2_P7_17	MT092684	Grp2_G4_14	MT093051
Grp1_P7_20	MT092149	Grp1_G6_18	MT092446	Grp2_P7_18	MT092685	Grp2_G4_15	MT093052
Grp1_P7_21	MT092150	Grp1_G6_19	MT092447	Grp2_P7_19	MT092686	Grp2_G4_16	MT093053
Grp1_P7_22	MT092151	Grp1_G6_20	MT092448	Grp2_P7_20	MT092687	Grp2_G4_17	MT093054
Grp1_P7_23	MT092152	Grp1_G6_21	MT092449	Grp2_P7_21	MT092688	Grp2_G4_18	MT093055
Grp1_P7_24	MT092153	Grp1_G6_22	MT092450	Grp2_P7_22	MT092689	Grp2_G4_19	MT093056

(Phylogroup 1) in (P)		(Phylogroup 1) in (G)		(Phylogroup 2) in (P)		(Phylogroup 2) in (G)	
Clone	Accession	Clone	Accession	Clone	Accession	Clone	Accession
Grp1_P7_25	MT092154	Grp1_G6_23	MT092451	Grp2_P7_23	MT092690	Grp2_G4_20	MT093057
Grp1_P7_26	MT092155	Grp1_G6_24	MT092452	Grp2_P7_24	MT092691	Grp2_G4_21	MT093058
Grp1_P7_27	MT092156	Grp1_G6_25	MT092453	Grp2_P7_25	MT092692	Grp2_G4_22	MT093059
Grp1_P7_28	MT092157	Grp1_G6_26	MT092454	Grp2_P7_26	MT092693	Grp2_G4_23	MT093060
Grp1_P7_29	MT092158	Grp1_G6_27	MT092455	Grp2_P7_27	MT092694	Grp2_G4_24	MT093061
Grp1_P7_30	MT092159	Grp1_G6_28	MT092456	Grp2_P7_28	MT092695	Grp2_G4_25	MT093062
Grp1_P2_1	MT092160	Grp1_G6_29	MT092457	Grp2_P7_29	MT092696	Grp2_G4_26	MT093063
Grp1_P2_2	MT092161	Grp1_G6_30	MT092458	Grp2_P7_30	MT092697	Grp2_G4_27	MT093064
Grp1_P2_3	MT092162	Grp1_G7_1	MT092459	Grp2_P7_31	MT092698	Grp2_G4_28	MT093065
Grp1_P2_4	MT092163	Grp1_G7_2	MT092460	Grp2_P7_32	MT092699	Grp2_G4_29	MT093066
Grp1_P2_5	MT092164	Grp1_G7_3	MT092461	Grp2_P7_33	MT092700	Grp2_G4_30	MT093067
Grp1_P2_6	MT092165	Grp1_G7_4	MT092462	Grp2_P7_34	MT092701	Grp2_G4_31	MT093068
Grp1_P2_7	MT092166	Grp1_G7_5	MT092463	Grp2_P7_35	MT092702	Grp2_G4_32	MT093069
Grp1_P2_8	MT092167	Grp1_G7_6	MT092464	Grp2_P7_36	MT092703	Grp2_G4_33	MT093070
Grp1_P2_9	MT092168	Grp1_G7_7	MT092465	Grp2_P7_37	MT092704	Grp2_G5_1	MT093071
Grp1_P2_10	MT092169	Grp1_G7_8	MT092466	Grp2_P7_38	MT092705	Grp2_G5_2	MT093072
Grp1_P2_11	MT092170	Grp1_G7_9	MT092467	Grp2_P7_39	MT092706	Grp2_G5_3	MT093073
Grp1_P2_12	MT092171	Grp1_G7_10	MT092468	Grp2_P7_40	MT092707	Grp2_G5_4	MT093074
Grp1_P2_13	MT092172	Grp1_G7_11	MT092469	Grp2_P7_41	MT092708	Grp2_G5_5	MT093075
Grp1_P2_14	MT092173	Grp1_G7_12	MT092470	Grp2_P7_42	MT092709	Grp2_G5_6	MT093076
Grp1_P2_15	MT092174	Grp1_G7_13	MT092471	Grp2_P7_43	MT092710	Grp2_G5_7	MT093077
Grp1_P2_16	MT092175	Grp1_G7_14	MT092472	Grp2_P7_44	MT092711	Grp2_G5_8	MT093078
Grp1_P2_17	MT092176	Grp1_G7_15	MT092473	Grp2_P7_45	MT092712	Grp2_G5_9	MT093079
Grp1_P2_18	MT092177	Grp1_G7_16	MT092474	Grp2_P7_46	MT092713	Grp2_G5_10	MT093080
Grp1_P2_19	MT092178	Grp1_G7_17	MT092475	Grp2_P2_1	MT092714	Grp2_G5_11	MT093081
Grp1_P2_20	MT092179	Grp1_G7_18	MT092476	Grp2_P2_2	MT092715	Grp2_G5_12	MT093082
Grp1_P2_21	MT092180	Grp1_G7_19	MT092477	Grp2_P2_3	MT092716	Grp2_G5_13	MT093083
Grp1_P2_22	MT092181	Grp1_G7_20	MT092478	Grp2_P2_4	MT092717	Grp2_G5_14	MT093084
Grp1_P2_23	MT092182	Grp1_G7_21	MT092479	Grp2_P2_5	MT092718	Grp2_G5_15	MT093085
Grp1_P2_24	MT092183	Grp1_G7_22	MT092480	Grp2_P2_6	MT092719	Grp2_G5_16	MT093086
Grp1_P2_25	MT092184	Grp1_G7_23	MT092481	Grp2_P2_7	MT092720	Grp2_G5_17	MT093087
Grp1_P2_26	MT092185	Grp1_G7_24	MT092482	Grp2_P2_8	MT092721	Grp2_G5_18	MT093088
Grp1_P2_27	MT092186	Grp1_G7_25	MT092483	Grp2_P2_9	MT092722	Grp2_G5_19	MT093089
Grp1_P2_28	MT092187	Grp1_G7_26	MT092484	Grp2_P2_10	MT092723	Grp2_G5_20	MT093090
Grp1_P2_29	MT092188	Grp1_G7_27	MT092485	Grp2_P2_11	MT092724	Grp2_G5_21	MT093091
Grp1_P2_30	MT092189	Grp1_G7_28	MT092486	Grp2_P2_12	MT092725	Grp2_G5_22	MT093092
Grp1_P8_1	MT092190	Grp1_G7_29	MT092487	Grp2_P2_13	MT092726	Grp2_G5_23	MT093093
Grp1_P8_2	MT092191	Grp1_G7_30	MT092488	Grp2_P2_14	MT092727	Grp2_G5_24	MT093094
Grp1_P8_3	MT092192	Grp1_G8_1	MT092489	Grp2_P2_15	MT092728	Grp2_G5_25	MT093095
Grp1_P8_4	MT092193	Grp1_G8_2	MT092490	Grp2_P2_16	MT092729	Grp2_G5_26	MT093096
Grp1_P8_5	MT092194	Grp1_G8_3	MT092491	Grp2_P2_17	MT092730	Grp2_G5_27	MT093097
Grp1_P8_6	MT092195	Grp1_G8_4	MT092492	Grp2_P2_18	MT092731	Grp2_G5_28	MT093098
Grp1_P8_7	MT092196	Grp1_G8_5	MT092493	Grp2_P2_19	MT092732	Grp2_G6_1	MT093099
Grp1_P8_8	MT092197	Grp1_G8_6	MT092494	Grp2_P2_20	MT092733	Grp2_G6_2	MT093100
Grp1_P8_9	MT092198	Grp1_G8_7	MT092495	Grp2_P2_21	MT092734	Grp2_G6_3	MT093101
Grp1_P8_10	MT092199	Grp1_G8_8	MT092496	Grp2_P2_22	MT092735	Grp2_G6_4	MT093102
Grp1_P8_11	MT092200	Grp1_G8_9	MT092497	Grp2_P2_23	MT092736	Grp2_G6_5	MT093103
Grp1_P8_12	MT092201	Grp1_G8_10	MT092498	Grp2_P2_24	MT092737	Grp2_G6_6	MT093104
Grp1_P8_13	MT092202	Grp1_G8_11	MT092499	Grp2_P2_25	MT092738	Grp2_G6_7	MT093105
Grp1_P8_14	MT092203	Grp1_G8_12	MT092500	Grp2_P2_26	MT092739	Grp2_G6_8	MT093106
Grp1_P8_15	MT092204	Grp1_G8_13	MT092501	Grp2_P2_27	MT092740	Grp2_G6_9	MT093107
Grp1_P8_16	MT092205	Grp1_G8_14	MT092502	Grp2_P2_28	MT092741	Grp2_G6_10	MT093108
Grp1_P8_17	MT092206	Grp1_G8_15	MT092503	Grp2_P2_29	MT092742	Grp2_G6_11	MT093109
Grp1_P8_18	MT092207	Grp1_G8_16	MT092504	Grp2_P2_30	MT092743	Grp2_G6_12	MT093110
Grp1_P8_19	MT092208	Grp1_G8_17	MT092505	Grp2_P2_31	MT092744	Grp2_G6_13	MT093111
Grp1_P8_20	MT092209	Grp1_G8_18	MT092506	Grp2_P2_32	MT092745	Grp2_G6_14	MT093112
Grp1_P8_21	MT092210	Grp1_G8_19	MT092507	Grp2_P2_33	MT092746	Grp2_G6_15	MT093113
Grp1_P8_22	MT092211	Grp1_G8_20	MT092508	Grp2_P8_1	MT092747	Grp2_G6_16	MT093114
Grp1_P8_23	MT092212	Grp1_G8_21	MT092509	Grp2_P8_2	MT092748	Grp2_G6_17	MT093115
Grp1_P8_24	MT092213	Grp1_G8_22	MT092510	Grp2_P8_3	MT092749	Grp2_G6_18	MT093116

(Phylogroup 1) in (P)		(Phylogroup 1) in (G)		(Phylogroup 2) in (P)		(Phylogroup 2) in (G)	
**Clone**	**Accession**	**Clone**	**Accession**	**Clone**	**Accession**	**Clone**	**Accession**
Grp1_P8_25	MT092214	Grp1_G8_23	MT092511	Grp2_P8_4	MT092750	Grp2_G6_19	MT093117
Grp1_P8_26	MT092215	Grp1_G8_24	MT092512	Grp2_P8_5	MT092751	Grp2_G6_20	MT093118
Grp1_P8_27	MT092216	Grp1_G8_25	MT092513	Grp2_P8_6	MT092752	Grp2_G6_21	MT093119
Grp1_P8_28	MT092217	Grp1_G8_26	MT092514	Grp2_P8_7	MT092753	Grp2_G6_22	MT093120
Grp1_P8_29	MT092218	Grp1_G8_27	MT092515	Grp2_P8_8	MT092754	Grp2_G6_23	MT093121
Grp1_P8_30	MT092219	Grp1_G8_28	MT092516	Grp2_P8_9	MT092755	Grp2_G6_24	MT093122
Grp1_P3_1	MT092220	Grp1_G8_29	MT092517	Grp2_P8_10	MT092756	Grp2_G6_25	MT093123
Grp1_P3_2	MT092221			Grp2_P8_11	MT092757	Grp2_G6_26	MT093124
Grp1_P3_3	MT092222			Grp2_P8_12	MT092758	Grp2_G6_27	MT093125
Grp1_P3_4	MT092223			Grp2_P8_13	MT092759	Grp2_G6_28	MT093126
Grp1_P3_5	MT092224			Grp2_P8_14	MT092760	Grp2_G6_29	MT093127
Grp1_P3_6	MT092225			Grp2_P8_15	MT092761	Grp2_G6_30	MT093128
Grp1_P3_7	MT092226			Grp2_P8_16	MT092762	Grp2_G7_1	MT093129
Grp1_P3_8	MT092227			Grp2_P8_17	MT092763	Grp2_G7_2	MT093130
Grp1_P3_9	MT092228			Grp2_P8_18	MT092764	Grp2_G7_3	MT093131
Grp1_P3_10	MT092229			Grp2_P8_19	MT092765	Grp2_G7_4	MT093132
Grp1_P3_11	MT092230			Grp2_P8_20	MT092766	Grp2_G7_5	MT093133
Grp1_P3_12	MT092231			Grp2_P8_21	MT092767	Grp2_G7_6	MT093134
Grp1_P3_13	MT092232			Grp2_P8_22	MT092768	Grp2_G7_7	MT093135
Grp1_P3_14	MT092233			Grp2_P8_23	MT092769	Grp2_G7_8	MT093136
Grp1_P3_15	MT092234			Grp2_P8_24	MT092770	Grp2_G7_9	MT093137
Grp1_P3_16	MT092235			Grp2_P8_25	MT092771	Grp2_G7_10	MT093138
Grp1_P3_17	MT092236			Grp2_P8_26	MT092772	Grp2_G7_11	MT093139
Grp1_P3_18	MT092237			Grp2_P8_27	MT092773	Grp2_G7_12	MT093140
Grp1_P3_19	MT092238			Grp2_P8_28	MT092774	Grp2_G7_13	MT093141
Grp1_P3_20	MT092239			Grp2_P8_29	MT092775	Grp2_G7_14	MT093142
Grp1_P3_21	MT092240			Grp2_P8_30	MT092776	Grp2_G7_15	MT093143
Grp1_P3_22	MT092241			Grp2_P8_31	MT092777	Grp2_G7_16	MT093144
Grp1_P3_23	MT092242			Grp2_P8_32	MT092778	Grp2_G7_17	MT093145
Grp1_P3_24	MT092243			Grp2_P8_33	MT092779	Grp2_G7_18	MT093146
Grp1_P3_25	MT092244			Grp2_P8_34	MT092780	Grp2_G7_19	MT093147
Grp1_P3_26	MT092245			Grp2_P8_35	MT092781	Grp2_G7_20	MT093148
Grp1_P3_27	MT092246			Grp2_P8_36	MT092782	Grp2_G7_21	MT093149
Grp1_P3_28	MT092247			Grp2_P8_37	MT092783	Grp2_G7_22	MT093150
Grp1_P3_29	MT092248			Grp2_P8_38	MT092784	Grp2_G7_23	MT093151
Grp1_P3_30	MT092249			Grp2_P8_39	MT092785	Grp2_G7_24	MT093152
Grp1_P9_1	MT092250			Grp2_P8_40	MT092786	Grp2_G7_25	MT093153
Grp1_P9_2	MT092251			Grp2_P8_41	MT092787	Grp2_G7_26	MT093154
Grp1_P9_3	MT092252			Grp2_P8_42	MT092788	Grp2_G7_27	MT093155
Grp1_P9_4	MT092253			Grp2_P8_43	MT092789	Grp2_G7_28	MT093156
Grp1_P9_5	MT092254			Grp2_P8_44	MT092790	Grp2_G7_29	MT093157
Grp1_P9_6	MT092255			Grp2_P8_45	MT092791	Grp2_G7_30	MT093158
Grp1_P9_7	MT092256			Grp2_P8_46	MT092792	Grp2_G8_1	MT093159
Grp1_P9_8	MT092257			Grp2_P8_47	MT092793	Grp2_G8_2	MT093160
Grp1_P9_9	MT092258			Grp2_P8_48	MT092794	Grp2_G8_3	MT093161
Grp1_P9_10	MT092259			Grp2_P8_49	MT092795	Grp2_G8_4	MT093162
Grp1_P9_11	MT092260			Grp2_P8_50	MT092796	Grp2_G8_5	MT093163
Grp1_P9_12	MT092261			Grp2_P3_1	MT092797	Grp2_G8_6	MT093164
Grp1_P9_13	MT092262			Grp2_P3_2	MT092798	Grp2_G8_7	MT093165
Grp1_P9_14	MT092263			Grp2_P3_3	MT092799	Grp2_G8_8	MT093166
Grp1_P9_15	MT092264			Grp2_P3_4	MT092800	Grp2_G8_9	MT093167
Grp1_P9_16	MT092265			Grp2_P3_5	MT092801	Grp2_G8_10	MT093168
Grp1_P9_17	MT092266			Grp2_P3_6	MT092802	Grp2_G8_11	MT093169
Grp1_P9_18	MT092267			Grp2_P3_7	MT092803	Grp2_G8_12	MT093170
Grp1_P9_19	MT092268			Grp2_P3_8	MT092804	Grp2_G8_13	MT093171
Grp1_P9_20	MT092269			Grp2_P3_9	MT092805	Grp2_G8_14	MT093172
Grp1_P9_21	MT092270			Grp2_P3_10	MT092806	Grp2_G8_15	MT093173
Grp1_P9_22	MT092271			Grp2_P3_11	MT092807	Grp2_G8_16	MT093174
Grp1_P9_23	MT092272			Grp2_P3_12	MT092808	Grp2_G8_17	MT093175
Grp1_P9_24	MT092273			Grp2_P3_13	MT092809	Grp2_G8_18	MT093176

(Phylogroup 1) in (P)		(Phylogroup 1) in (G)		(Phylogroup 2) in (P)		(Phylogroup 2) in (G)	
Clone	Accession	Clone	Accession	Clone	Accession	Clone	Accession
Grp1_P9_25	MT092274			Grp2_P3_14	MT092810	Grp2_G8_19	MT093177
Grp1_P9_26	MT092275			Grp2_P3_15	MT092811	Grp2_G8_20	MT093178
Grp1_P9_27	MT092276			Grp2_P3_16	MT092812	Grp2_G8_21	MT093179
Grp1_P9_28	MT092277			Grp2_P3_17	MT092813	Grp2_G8_22	MT093180
Grp1_P9_29	MT092278			Grp2_P3_18	MT092814	Grp2_G8_23	MT093181
				Grp2_P3_19	MT092815	Grp2_G8_24	MT093182
				Grp2_P3_20	MT092816	Grp2_G8_25	MT093183
				Grp2_P3_21	MT092817	Grp2_G8_26	MT093184
				Grp2_P3_22	MT092818		
				Grp2_P3_23	MT092819		
				Grp2_P3_24	MT092820		
				Grp2_P3_25	MT092821		
				Grp2_P3_26	MT092822		
				Grp2_P3_27	MT092823		
				Grp2_P3_28	MT092824		
				Grp2_P3_29	MT092825		
				Grp2_P3_30	MT092826		
				Grp2_P3_31	MT092827		
				Grp2_P3_32	MT092828		
				Grp2_P3_33	MT092829		
				Grp2_P3_34	MT092830		
				Grp2_P3_35	MT092831		
				Grp2_P3_36	MT092832		
				Grp2_P9_1	MT092833		
				Grp2_P9_2	MT092834		
				Grp2_P9_3	MT092835		
				Grp2_P9_4	MT092836		
				Grp2_P9_5	MT092837		
				Grp2_P9_6	MT092838		
				Grp2_P9_7	MT092839		
				Grp2_P9_8	MT092840		
				Grp2_P9_9	MT092841		
				Grp2_P9_10	MT092842		
				Grp2_P9_11	MT092843		
				Grp2_P9_12	MT092844		
				Grp2_P9_13	MT092845		
				Grp2_P9_14	MT092846		
				Grp2_P9_15	MT092847		
				Grp2_P9_16	MT092848		
				Grp2_P9_17	MT092849		
				Grp2_P9_18	MT092850		
				Grp2_P9_19	MT092851		
				Grp2_P9_20	MT092852		
				Grp2_P9_21	MT092853		
				Grp2_P9_22	MT092854		
				Grp2_P9_23	MT092855		
				Grp2_P9_24	MT092856		
				Grp2_P9_25	MT092857		
				Grp2_P9_26	MT092858		
				Grp2_P9_27	MT092859		
				Grp2_P9_28	MT092860		
				Grp2_P9_29	MT092861		
				Grp2_P9_30	MT092862		
				Grp2_P9_31	MT092863		
				Grp2_P9_32	MT092864		
				Grp2_P9_33	MT092865		
				Grp2_P9_34	MT092866		
				Grp2_P9_35	MT092867		
				Grp2_P9_36	MT092868		
				Grp2_P9_37	MT092869		

(Phylogroup 1) in (P)		(Phylogroup 1) in (G)		(Phylogroup 2) in (P)		(Phylogroup 2) in (G)	
Clone	Accession	Clone	Accession	Clone	Accession	Clone	Accession
				Grp2_P9_38	MT092870		
				Grp2_P9_39	MT092871		
				Grp2_P9_40	MT092872		
				Grp2_P9_41	MT092873		
				Grp2_P9_42	MT092874		
				Grp2_P9_43	MT092875		
				Grp2_P9_44	MT092876		
				Grp2_P9_45	MT092877		
				Grp2_P9_46	MT092878		
				Grp2_P9_47	MT092879		
				Grp2_P9_48	MT092880		
				Grp2_P9_49	MT092881		
				Grp2_P9_50	MT092882		
				Grp2_P9_51	MT092883		
				Grp2_P9_52	MT092884		

(Phylogroup 1) in (P): Name of treponeme phylogroup 1 *pyrH* genes identified in periodontitis (P) subjects. **(Phylogroup 1) in (G)**: Name of treponeme phylogroup 1 *pyrH* genes identified in gingivitis (G) subjects. **(Phylogroup 2) in (P)**: Name of treponeme phylogroup 2 *pyrH* genes identified in periodontitis (P) subjects. **(Phylogroup 2) in (G)**: Name of treponeme phylogroup 2 *pyrH* genes identified in periodontitis (G) subjects. The identity of the clone is indicated, e.g. Grp1_P1_1 refers to the first clone of oral treponeme phylogroup 1 *pyrH* gene sequence from patient P1.
